# Sample Size Requirements for Applying Diagnostic Classification Models

**DOI:** 10.3389/fpsyg.2020.621251

**Published:** 2021-01-25

**Authors:** Sedat Sen, Allan S. Cohen

**Affiliations:** ^1^Educational Sciences Department, Faculty of Education, Harran University, Sanliurfa, Turkey; ^2^Educational Psychology Department, College of Education, University of Georgia, Athens, GA, United States

**Keywords:** diagnostic classification models, cognitive diagnostic models, sample size, item recovery, classification accuracy

## Abstract

Results of a comprehensive simulation study are reported investigating the effects of sample size, test length, number of attributes and base rate of mastery on item parameter recovery and classification accuracy of four DCMs (i.e., C-RUM, DINA, DINO, and LCDMREDUCED). Effects were evaluated using bias and RMSE computed between true (i.e., generating) parameters and estimated parameters. Effects of simulated factors on attribute assignment were also evaluated using the percentage of classification accuracy. More precise estimates of item parameters were obtained with larger sample size and longer test length. Recovery of item parameters decreased as the number of attributes increased from three to five but base rate of mastery had a varying effect on the item recovery. Item parameter and classification accuracy were higher for DINA and DINO models.

## Introduction

Diagnostic classification models (DCMs), also known as cognitive diagnostic models (CDMs), can be viewed as restricted versions of general latent class models (Rupp and Templin, 2008). These models provide one way of classifying respondents into different diagnostic states. They are computationally intensive and generally require use of iterative algorithms to obtain estimates of model parameters. Both general and specific DCMs have been proposed in the educational and psychological measurement literature. Examples of Specific DCMs include deterministic inputs, noisy “and” gate (DINA; Haertel, 1989; Junker and Sijtsma, 2001), deterministic inputs, noisy “or” gate (DINO; Templin and Henson, 2006), noisy-input, deterministic “and” gate (NIDA), and the compensatory reparameterized unified model (C-RUM; Hartz, 2002). General DCMs include the log-linear cognitive diagnostic model (LCDM; Henson et al., 2009), the general diagnostic model (GDM; von Davier, 2005), and the generalized DINA (G-DINA; de la Torre, 2011) model. de la Torre (2011) and von Davier (2014) have shown that these three general models are equivalent.

The LCDM specifies the conditional probability that examinee *j* with attribute pattern α_c_ provides a correct answer to item *i* as

(1) $$\begin{array}{l} P\left(X_{ic}=1|\alpha_{\text{c}}\right)=\frac{\exp\left(\lambda_{i,0}+\lambda_{\text{i}}^{\text{T}}h\left(\alpha_{\text{c}},{\text{q}}_{i}\right)\right)}{1+\exp\left(\lambda_{i,0}+\lambda_{\text{i}}^{\text{T}}h\left(\alpha_{\text{c}},{\text{q}}_{i}\right)\right)} \end{array}$$

where λ_*i*, 0_ represents the intercept, that is the logit of a correct response for an examinee who has not mastered any of the attributes required by item *i*. ${\text{λ}}_{i}^{T}h\left(\alpha_{\text{c}},q_{i}\right)$ is the kernel function as shown below:

(2) $$\begin{array}{l} \lambda_{\text{i}}^{\text{T}}h\left(\alpha_{c},{\text{q}}_{i}\right)=\overset{A}{\underset{a=1}{\sum }}\lambda_{i,1,\left(a\right)}\alpha_{ca}q_{ia}+\overset{A-1}{\underset{a=1}{\sum }}\overset{A}{\underset{a^{\prime }>1}{\sum }}\lambda_{i,2,\left(a,a^{\prime }\right)\alpha_{ca}\alpha_{ca^{\prime }}q_{ia}q_{ia^{\prime }}+\ldots \;} \end{array}$$

The **q**_*i*_ represents the *i*th row vector of the Q-matrix (Tatsuoka, 1983) that consists of 0 and 1 to indicate an item *i* gives information about the presence of an attribute *a* (*a* = 1, …, A). That is, **q**_*ia*_ = 1 when item *i* requires attribute *a* for correct response and 0 otherwise. The vector $\alpha_{\text{c}}={\left(\alpha_{\text{c}1},\ldots ,\alpha_{\text{cA}}\right)}^{\text{T}}$ includes the attribute mastery pattern that belongs to latent class *c*. The total number of possible latent classes in a DCM equals 2^A^ where A is the number of attributes that should be mastered to respond to an item correctly. For instance, an item requiring three attributes yields eight latent classes. A full LCDM can have different item parameters including intercept [e.g., λ_1,0_], main [e.g., λ_1,1(2)_], and interaction [e.g., λ_1,2(3,4)_]. Other DCMs such as DINA, DINO, and C-RUM models can be obtained from the full LCDM (see Equation 1) by modifying different parameter constraints.

Suppose that a number of items measuring arithmetic ability (addition, subtraction, multiplication, and division) were included on the test. Some of the items may require only addition (Attribute 1), and some may require only subtraction (Attribute 2). However, some of the items can require two attributes (multiplication and division) at the same time. Suppose Item 1 (for example, 5 × 8/4 =) requires having two attributes: multiplication (Attribute 3) and division (Attribute 4). The item response function for Item 1 can be written as

(3) $$\begin{array}{l} \text{P}\left({\text{X}}_{1\text{c}}=1|\alpha_{\text{c}3},\alpha_{\text{c}4}\right)\\ =\frac{\text{exp}\left({\text{λ}}_{1,0}+\lambda_{1,1\left(3\right)}\alpha_{\text{c}3}+\lambda_{1,1\left(4\right)}\alpha_{\text{c}4}+\lambda_{1,2\left(3,4\right)}\alpha_{\text{c}3}\alpha_{\text{c}4}\right)}{1+\text{exp}\left(\lambda_{1,0}+\lambda_{1,1\left(3\right)}\alpha_{\text{c}3}+\lambda_{1,1\left(4\right)}\alpha_{\text{c}4}+\lambda_{1,2\left(3,4\right)}\alpha_{\text{c}3}\alpha_{\text{c}4}\right)}, \end{array}$$

where α_*c*3_ and α_*c*4_ are latent variables for Attribute 3 and 4, respectively. This model includes one intercept (λ_1,0_), two main effects [λ_1,1(3)_ and λ_1,1(4)_], and a two-way interaction effect [λ_1,2(3,4)_] between two attributes. The latent predictor variables are combined by a series of linear modeling effects that can result in compensatory or non-compensatory DCMs. These specific DCMs do not include all of the terms in the item response function. Each DCM makes different assumptions about mastery of attributes and their effects on the item response. For example, the DINA model requires mastery of both attributes to be able to correctly respond to this item. Thus, the item response function for Item 1 includes an intercept and a two-way interaction term as shown below:

(4) $$\begin{array}{l} \text{P}\left({\text{X}}_{1\text{c}}=1|\alpha_{\text{c}3},\alpha_{\text{c}4}\right)=\frac{\text{exp}\left({\text{λ}}_{1,0}+\lambda_{1,2\left(3,4\right)}\alpha_{\text{c}3}\alpha_{\text{c}4}\right)}{1+\text{exp}\left(\lambda_{1,0}+\lambda_{1,2\left(3,4\right)}\alpha_{\text{c}3}\alpha_{\text{c}4}\right)} \end{array}$$

The DINO model, on the other hand, functions differently than the DINA, as it requires the mastery of at least one attribute for a correct response. Students mastering either Attribute 3 or Attribute 4 can get this item correct. Thus, the item response function for this item can be written as

(5) $$\begin{array}{l} \text{P}\left({\text{X}}_{1\text{c}}=1|\alpha_{\text{c}3},\alpha_{\text{c}4}\right)=\frac{\exp\left(\lambda_{1,0}+\lambda_{\text{aA}}\right)}{1+\exp\left(\lambda_{1,0}+\lambda_{\text{A}}\right)} \end{array}$$

where λ_*A*_ = λ_1,1(3)_α_*c*3_ + λ_1,1(4)_α_*c*4_ − λ_1,2(3,4)_α_*c*3_α_*c*4_ (see also Rupp et al., 2010, p. 163) As shown in the equation, the DINO model includes two parameters (λ_1,0_ and λ_*A*_). Finally, consider Item 1 for the C-RUM. The C-RUM can be considered an LCDM without an interaction effect as it includes only intercept and main effects as shown below:

(6) $$\begin{array}{l} \text{P}\left({\text{X}}_{1\text{c}}=1|\alpha_{\text{c}3},\alpha_{\text{c}4}\right)=\frac{\text{exp}\left({\text{λ}}_{1,0}+\lambda_{1,1\left(3\right)}\alpha_{\text{c}3}+\lambda_{1,1\left(4\right)}\alpha_{\text{c}4}\right)}{1+\text{exp}\left(\lambda_{1,0}+\lambda_{1,1\left(3\right)}\alpha_{\text{c}3}+\lambda_{1,1\left(4\right)}\alpha_{\text{c}4}\right)} \end{array}$$

Slipping and guessing parameters are typically used in DCMs to describe item characteristics. Slipping parameter is used for a situation when a respondent who mastered all the required attributes for an item but fails to answer the item correctly, and guessing parameter refers to a situation when a respondent who lacks at least one of the required attributes for an item correctly answers the item. These parameters can be obtained using the intercept, main, and interaction terms presented in Equations (3)–(6). For instance, $\frac{\exp\left(\lambda_{1,0}\right)}{1+\exp\left(\lambda_{1,0}\right)}$ can be used to estimate guessing (*g*_*i*_) and $\frac{\exp\left(\lambda_{1,0}+\lambda_{1,2\left(3,4\right)}\right)}{1+\exp\left(\lambda_{1,0}+\lambda_{1,2\left(3,4\right)}\right)}$ to estimate the one minus slipping (1 – *s*_*i*_) parameter in DINA model (Rupp et al., 2010). Slipping parameter can be defined differently for other DCMs (see also Rupp et al., 2010).

As is the case with many statistical models, model parsimony is an important consideration in DCM selection, such that the simpler model is generally preferred over the more complex model. More complex DCMs require larger sample sizes to yield accurate estimates and more reduced DCMs can usually be estimated accurately with smaller sample sizes. Reduced models also typically provide for more straightforward interpretations and higher correct classification rates than more saturated models, particularly when the sample sizes are small.

Complexities which require larger samples tend to increase with the numbers of attributes and items. Sessoms and Henson (2018) have shown that 61% of the studies of DCMs have used sample sizes >1,000 and 31% have used sample sizes of 1,000–2,000. Some research has been reported with samples as small as 44 (Jang et al., 2015) and 96 (Im and Yin, 2009). Results of these latter studies have been reported with low or negative item discrimination values.

One concern with respect to sample size is that there is as yet little information reported on use of DCMs with smaller samples although a number of studies have been conducted on the effect of sample sizes on different aspects of diagnostic models. For instance, Akbay (2016) showed that the non-parametric cognitive diagnosis approach (Chiu and Douglas, 2013) performs as well as the CDM based empirical Bayes estimation method for attribute classification in the presence of small sample sizes such as 250, 500, and 1,000. Sünbül and Kan (2016) investigated the effect of several factors including number of attributes and sample size (i.e., 200, 500, 1,000, and 5,000) on model fit, item recovery, and classification accuracy of the DINA model. The number of attributes and sample size had positive effects on the model estimates. Lei and Li (2016) investigated the performance of several model-fit indices for selecting model and on Q-matrix design under four sample size levels (500, 1,000, 2,000, and 4,000). Results indicated that performance of fit indices appeared to increase as the sample size increased. Tzou and Yang (2019) also compared the performance of model fit indices in CDMs using small sample sizes (i.e., 50, 75, 100, and 200) and showed that AIC (Akaike, 1974) performed better than other indices. Similarly, Hu et al. (2016) evaluated model fit for CDMs using sample sizes of 200, 500, and 1,000 and showed that performances of the three relative fit statistics AIC, BIC (Schwarz, 1978), and CAIC (Bozdogan, 1987) improved when sample size increased. Başokcu (2014) found classification accuracy increased as the number of attributes (1–5) decreased and sample size increased in DINA and G-DINA models. Similarly de la Torre et al. (2010) showed that sample size increase from 1,000 to 4,000 reduced the bias in item parameter estimates.

In another sample size related simulation study, Cui et al. (2012) showed that the asymptotic normal theory of classification consistency index and classification accuracy index can be applied with small sample sizes (100, 500 and 1,000) for attribute classifications in DINA model. Paulsen (2019) also investigated the effect of very small sample sizes including 25, 50, 150, and 1,000 simulated respondents on three CDMs (DINA, non-parametric cognitive diagnosis, and the supervised artificial neural network models) by focusing on characteristics such as model performances and model fit. Results of that study showed that those three models were able to estimate examinee classifications at even the smallest sample size. Galeshi and Skaggs (2016) conducted a simulation study using C-RUM under different sample size levels including 50, 100, 500, 1,000, 5,000, and 10,000, and showed that attribute classification was effected by different combinations of sample size and test length.

Choi et al. (2010) found that relative model fit indices were able to detect the correct DCM with samples of 200 or more. Rojas et al. (2012) found attribute classification accuracy of DINA, DINO, A-CDM, and G-DINA models was more accurate when test length and sample size were large. Previous simulation studies have focused on a limited number of models under the assumption that test items measured a common underlying model. However, this may not necessarily be the case as each item may also be designed to reflect a specific DCM. Thus, a more comprehensive simulation study is needed to examine the effects of small sample size on the classification accuracy and on parameter estimates when the test items reflect different model structures.

The present study was designed to investigate the effects of sample size on estimation and accuracy of parameter estimates and on classification as a function of sample size and for different types of DCMs. This study investigated the performance of specific DCMs under a set of practical testing conditions.

## Materials and Methods

### Simulation Study Design

Parameter recovery and classification accuracy for the LCDMs were assessed under several simulated conditions. In this regard, five factors were manipulated: sample size (50, 100, 200, 300, 400, 500, 1,000, and 5,000), test length (12, 24, and 36 items), number of attributes (3 and 5), base rate (0.25 and 0.50), and generating model (the reduced LCDM, the DINA model, the DINO model, and the C-RUM). A total of 100 replications were simulated for each condition using the maximum likelihood estimation algorithm as implemented in the Mplus 8.4 software package (Muthén and Muthén, 1998–2019).

### Constant Factors

For purposes of this study, tetrachoric correlation between each pair of attributes, item quality, and Q-matrices were held constant across simulation conditions. The tetrachoric correlation between each pair of attributes was set to be 0.70. This is within the typical range of correlations for subdomains in national and international educational assessments (Kunina-Habenicht et al., 2012).

Medium level item quality was used to simulate items that were better at separating masters and non-masters of the measured attributes. This was achieved with medium level item discrimination based on the difference in the probability of a correct response for two groups of students (i.e., item discrimination value of 0.60 = 0.85–0.25). In this study, two different Q-matrix specifications were used for models with three and five attributes. The Q-matrix specification used in this study was intended to reflect the kinds of Q-matrices used in previous simulation studies with DCMs (e.g., Kunina-Habenicht et al., 2012; de la Torre and Chiu, 2016). The Q-matrix of the models with three attributes and 36 items used in this study is presented in Table 1. The first 12 rows of the Q-matrix in Table 1 were used for the 12-item models and the first 24 rows of the Q-matrix in Table 1 were used for the 24-item models. The Q-matrix of the models with 5 attributes and 36 items is presented in Table 2. The 12-item and 24-item models include the first 12 and 24 rows of this table, respectively.

**Table 1:** Q-Matrix for conditions with three attributes.

	**Attribute**					**Attribute**			
**Item**	**1**	**2**	**3**	Item type^*^	**Item**	**1**	**2**	**3**	Item type^*^
1	1	0	0	LCDM^**^	19	1	0	0	DINO^**^
2	0	1	0	DINA^**^	20	0	1	0	C-RUM^**^
3	0	0	1	DINO^**^	21	0	0	1	LCDM^**^
4	1	1	0	C-RUM	22	1	1	0	DINA
5	1	0	1	LCDM	23	1	0	1	DINO
6	0	1	1	DINA	24	0	1	1	C-RUM
7	1	0	0	DINO^**^	25	1	0	0	LCDM^**^
8	0	1	0	C-RUM	26	0	1	0	DINA^**^
9	0	0	1	LCDM^**^	27	0	0	1	DINO^**^
10	1	1	0	DINA	28	1	1	0	C-RUM
11	1	0	1	DINO	29	1	0	1	LCDM
12	0	1	1	C-RUM	30	0	1	1	DINA
13	1	0	0	LCDM^**^	31	1	0	0	DINO^**^
14	0	1	0	DINA	32	0	1	0	C-RUM^**^
15	0	0	1	DINO^**^	33	0	0	1	LCDM^**^
16	1	1	0	C-RUM	34	1	1	0	DINA
17	1	0	1	LCDM	35	1	0	1	DINO
18	0	1	1	DINA	36	0	1	1	C-RUM

* *Item type specified for LCDMREDUCED model across all items*.

** *The parameterization of four DCMs are the same for items requiring only 1 attribute*.

**Table 2:** Q-Matrix for conditions with five attributes.

	**Attribute**						**Attribute**				
**Item**	**1**	**2**	**3**	**4**	**5**	**Item**	**1**	**2**	**3**	**4**	**5**
1	1	0	0	0	0	19	1	0	1	0	0
2	0	1	0	0	0	20	1	0	0	1	0
3	0	0	1	0	0	21	1	0	0	0	1
4	0	0	0	1	0	22	0	1	1	0	0
5	0	0	0	0	1	23	0	1	0	1	0
6	1	1	0	0	0	24	0	1	0	0	1
7	1	0	1	0	0	25	1	0	0	0	0
8	1	0	0	1	0	26	0	1	0	0	0
9	1	0	0	0	1	27	0	0	1	0	0
10	0	1	1	0	0	28	0	0	0	1	0
11	0	1	0	1	0	29	0	0	0	0	1
12	0	1	0	0	1	30	1	1	0	0	0
13	1	0	0	0	0	31	1	0	1	0	0
14	0	1	0	0	0	32	1	0	0	1	0
15	0	0	1	0	0	33	1	0	0	0	1
16	0	0	0	1	0	34	0	1	1	0	0
17	0	0	0	0	1	35	0	1	0	1	0
18	1	1	0	0	0	36	0	1	0	0	1

### Manipulated Factors

The simulation study had five manipulated factors including sample size, test length, number of attributes, base rate, and generating models. The sample sizes were 50, 100, 200, 300, 400, 500, 1,000, and 5,000 simulated examinees. These values were selected to represent a range of sample sizes from very small (50) to large (5,000). The number of respondents were selected to comply with studies reported in the DCM literature. Rojas et al. (2012), for example, used 100, 200, 400, 800, and 1,600 and Başokcu (2014) used 30, 50, 100, 200, and 400 for the sample size conditions. In this study, we extended sample sizes to include 5,000 simulated examinees.

Test length included 12, 24, and 36 items. These were intended to simulate small, medium, and long test lengths. For a math test, for example, it usually takes about 1 ½ min per multiple-choice item. For a 36-item test, this would actually be 48 min, which would be a relatively long amount of time for most students up to and including high school age. These test lengths were set to be multiples of four to produce items based on four different models in the reduced LCDM model. The numbers of items included in this study are typical of test lengths observed in real tests such as the TIMSS and PISA tests. For example, TIMSS assessment items are grouped into a series of item blocks, with ~10–14 items in each block at the fourth grade and 12–18 items at the eighth grade level. Similarly, von Davier et al. (2019) notes that the number of items administered in each assessment cycle of PISA consisted of 28 items for reading in 2003 and 35 items for mathematics assessment in 2009.

The numbers of attributes were three and five to reflect numbers commonly found in educational and psychological tests (Kunina-Habenicht et al., 2012). For instance, Chen and Chen (2016) reported a Q-matrix with five attributes by employing the five processes (skills) of reading under the PISA assessment framework. Examples of simulation or real data studies with three or five attributes include de la Torre and Douglas (2004), de la Torre (2009), de la Torre and Lee (2010), Kunina-Habenicht et al. (2012), Templin and Bradshaw (2014), de la Torre and Chiu (2016), Hu et al. (2016), and Sen and Bradshaw (2017). The base rate of mastery for an attribute is the proportion of examinees who have mastered the attribute in the population. This was set to 0.25 and 0.50. A base rate of 0.50 is commonly reported in the literature (e.g., Kunina-Habenicht et al., 2012; Bradshaw and Madison, 2016; Sen and Bradshaw, 2017). As in Sen and Bradshaw (2017), the 0.25 base rate condition was added to investigate item recovery and classification accuracy comparisons under less optimal conditions. Base rate mastery and tetrachoric correlations between each pair of attributes were generated using the SAS macro created by Templin and Hoffman (2013). The SAS code used to generate the 0.25 base rate is presented in the Supplementary Data.

Four different data-generating models were simulated, including (a) Reduced LCDM, (b) C-RUM, (c) DINA, and (d) DINO. For the reduced LCDM, the underlying DCM structures were generated to differ across the complex items with the following common sub-models of the LCDM. For a 12-item test, three complex items were generated under the DINA model, three under the DINO model, three under the C-RUM, and three under the saturated LCDM (see Table 1). The specific item structures used in LCDMREDUCED model for 12-, 24-, and 36-item tests are presented in Table 1. For the DINA, DINO, and C-RUM models, the underlying DCM structure was generated to be the same. That is, all of the items were of a common type. The same patterns were used for the 5-attribute matrix presented in Table 2.

### Data Generation

Data generation was done using Mplus. First, a full saturated LCDM syntax was created using *MplusDCM_functions.R* function with the MplusAutomation (Hallquist and Wiley, 2018) package in R. The *MplusDCM_functions.R* function created by Andre Rupp and Oliver Wilhelm was used to build, run, and parse Mplus syntax for estimation of the LCDM. After generating the full LCDM syntax using this function, the syntax for specific DCMs was created by modifying the syntax using the descriptions provided by Rupp et al. (2010) and Sen and Terzi (2020). The LCDM syntax for Item 4 from Table 1, for example, included an intercept (λ_4,0_), two main effects [λ_4,1(1)_ and λ_4,1(2)_] for Attribute 1 and Attribute 2 and an interaction effect [λ_4,2(1,2)_] between these two attributes. In this example, 3 attributes yield 8 attribute patterns or classes. Then, the LCDM kernel (see Equation 2) was specified for each class and each item to assign latent classes to an attribute pattern or profile. Finally, unique item response functions for each item were specified in the Item-to-Profile table (see Table 3). The following labels used in the Mplus code as shown in Table 3 are t4_1, t4_2, t4_3, and t4_4. These were created using the Item-to-Profile table.

**Table 3:** Item-to-profile table for item 4.

	**c_**1**_**	**c_**2**_**	**c_**3**_**	**c_**4**_**	**c_**5**_**	**c_**6**_**	**c_**7**_**	**c_**8**_**
**α**_**c**_	[0,0,0]	[0,0,1]	[0,1,0]	[0,1,1]	[1,0,0]	[1,0,1]	[1,1,0]	[1,1,1]
LCDM	λ_4,0_	λ_4,0_	λ_4,0_ + λ_4,1(2)_	λ_4,0_ + λ_4,1(2)_	λ_4,0_ + λ_4,1(1)_	λ_4,0_ + λ_4,1(1)_	λ_4,0_ + λ_4,1(1)_ + λ_4,1(2)_ + λ_4,2(1,2)_	λ_4,0_ + λ_4,1(1)_ + λ_4,1(2)_ + λ_4,2(1,2)_
Mplus label	t4_1	t4_1	t4_2	t4_2	t4_3	t4_3	t4_4	t4_4

The specific part of the Mplus syntax for LCDM is presented in the first column of Table 4. As can be seen in the first column, the LCDM syntax to estimate intercept, main effects, and the interaction term is labeled as l4_0, l4_12, l4_11, and l4_212, respectively. C-RUM, DINA, and DINO models are presented in the next three columns of Table 4. The C-RUM does not include any interaction effect. The DINA and DINO models only include intercept and *e* parameters. The Mplus syntax for these latter two models are not the same, however, due to differences in the kernel function.

**Table 4:** Mplus syntax specifications for item 4 (Q-matrix entry 110).

**Full LCDM**	**C-RUM**	**DINA**	**DINO**
NEW (l4_0 l4_12 l4_11 l4_212);	NEW (l4_0 l4_12 l4_11);	NEW (l4_0 l4_e);	NEW (l4_0 l4_e);
t4_1 = –(l4_0);	t4_1 = –(l4_0);	t4_1 = –(l4_0);	t4_1 = –(l4_0);
t4_2 = –(l4_0 + l4_12);	t4_2 = –(l4_0 + l4_12);	t4_2 = –(l4_0);	t4_2 = –(l4_0 + l4_e);
t4_3 = –(l4_0 + l4_11);	t4_3 = –(l4_0 + l4_11);	t4_3 = –(l4_0);	t4_3 = –(l4_0 + l4_e);
t4_4 = –(l4_0 + l4_11 + l4_12 + l4_212);	t4_4 = –(l4_0 + l4_11 + l4_12);	t4_4 = –(l4_0 + l4_e);	t4_4 = –(l4_0 + l4_e);
! Order constrains	! Order constrains	! Order constrains	! Order constrains
l4_12 > 0;	l4_12 > 0;	l4_e > 0;	l4_e > 0;
l4_11 > 0;	l4_11 > 0;		
l4_212 > –l4_11;			
l4_212 > –l4_12;			

The MONTECARLO command in Mplus was used to generate 100 data sets for each condition. The true generating values for intercept, main, *e* parameter, and interaction effects were set to be −1.1, 1.3, 3, and 0.24, respectively. These values were selected to produce items with medium-quality with an item discrimination value of 0.60. The item discrimination is specified by taking the difference in the probability of a correct response for two groups of students (0.60 = 0.85–0.25).

### Estimation

All of the models were estimated using maximum likelihood estimation (i.e., MLR) as implemented in Mplus. The following Mplus options were used to obtain estimates of model parameters: ANALYSIS: TYPE = MIXTURE; STARTS = 200 20; PROCESSORS = 8;. Eight classes and 32 classes were modeled for the 3-Attribute and 5-Attribute models in the MODEL command using the labels from Table 3. The MODEL CONSTRAINT part in Mplus syntax was modified using the model constraints described in Table 4. The vectors of attribute classifications were obtained based on expected a posteriori estimation by specifying the FILE = “respondents#.cprob”; option under SAVEDATA command in Mplus. In total, 38,400 Mplus analyses (8 × 3 × 2 × 4 × 2 × 100) were run using a Linux (64-bit Centos 7) high performance computing (HPC) cluster. Item parameter estimates for intercept, main, *e* parameter, and interaction effects and class probability values were extracted from each Mplus files using MplusAutomation package.

### Evaluation Criteria

Recovery of item parameters were assessed using the root mean square error (RMSE) and bias across replications. RMSE and bias values for intercept, main, *e* parameter, and interaction terms were calculated using the following formulas:

(7) $$\begin{array}{l} \text{RMSE}=\sqrt{\frac{\sum_{\text{r}=1}^{\text{R}}{\left(\lambda_{\text{i}}-{\hat{\lambda }}_{\text{ir}}\right)}^{2}}{\text{R}}}, \end{array}$$

(8) $$\begin{array}{l} \text{Bias}=\frac{\sum_{\text{r}=1}^{\text{R}}\left(\lambda_{\text{i}}-{\hat{\lambda }}_{\text{ir}}\right)}{\text{R}}, \end{array}$$

where *R* is the total number of replications (*r* = 1, … *R*) and λ_*i*_ is the true parameter value of intercept, main, *e* parameter or interaction term for item *i*. ${\hat{\lambda }}_{ir}$ refers to estimated item parameters for item *i* under the *r*th replication. Classification accuracy of attribute profiles under each condition was determined by calculating the percentage of examinees whose estimated attribute profile was the same as the simulated (i.e., true) attribute profile.

## Results

**Figure 1:**
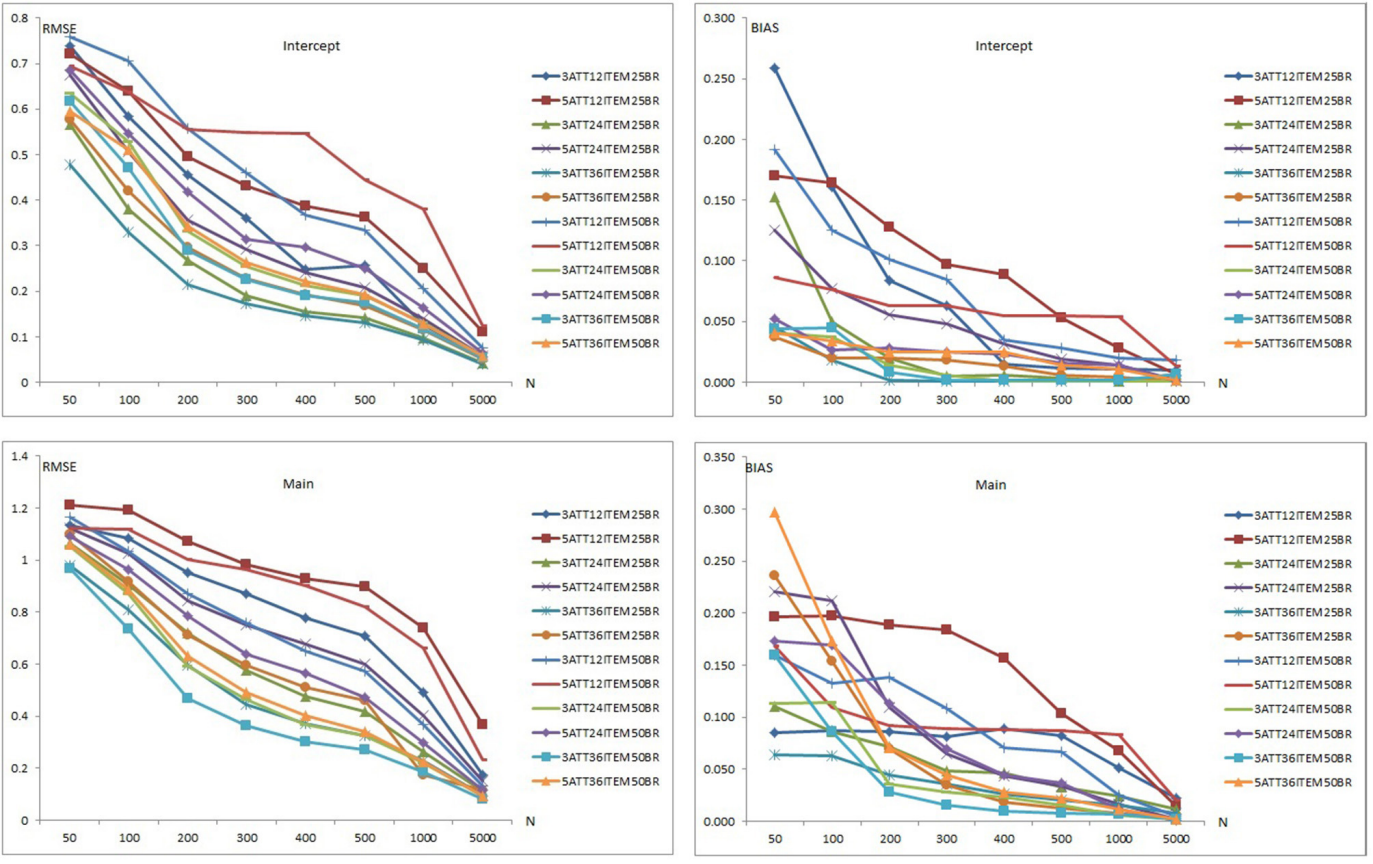
Mean RMSE and bias plots for the C-RUM.

**Figure 2:**
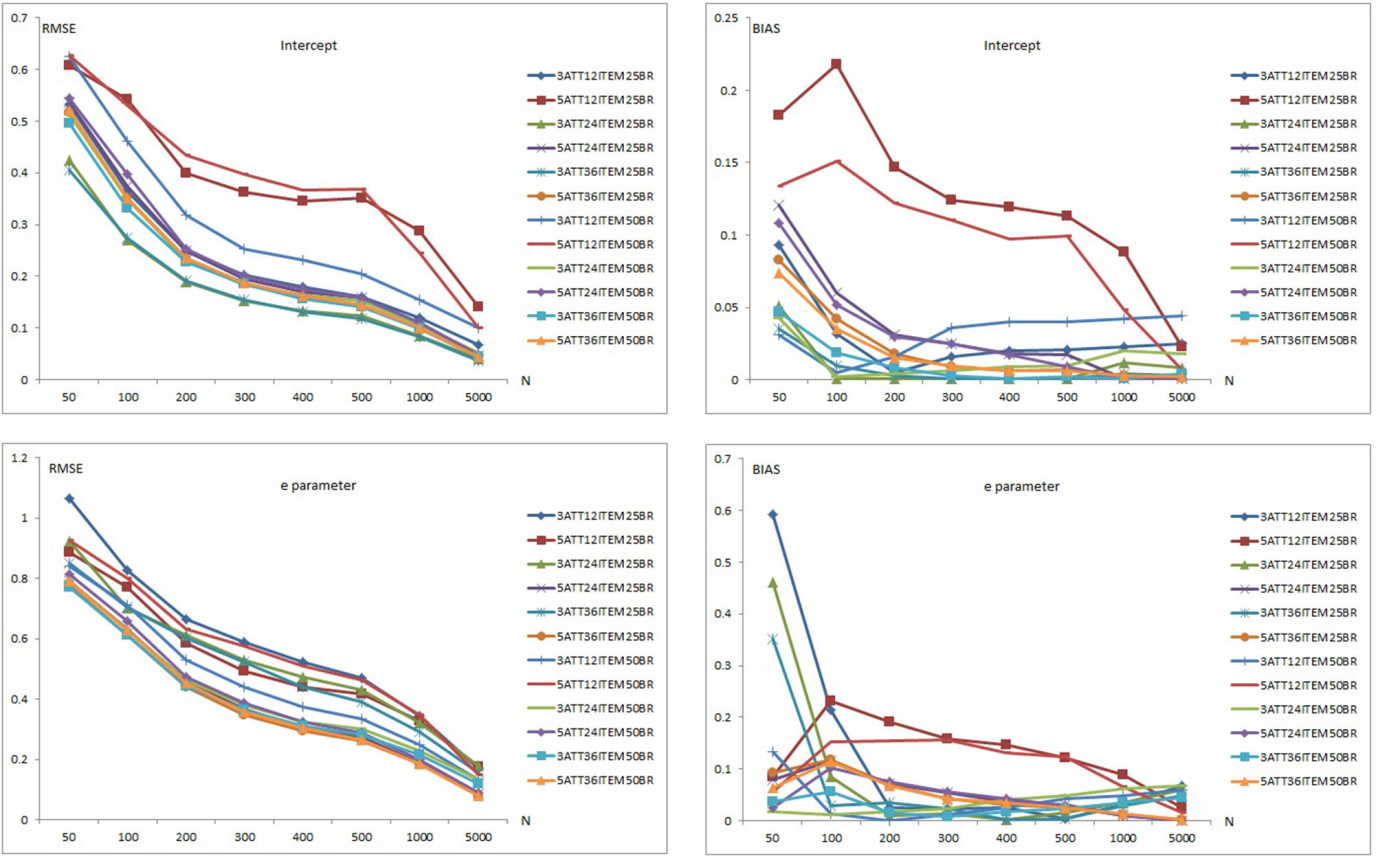
Mean RMSE and bias plots for the DINA Model.

**Figure 3:**
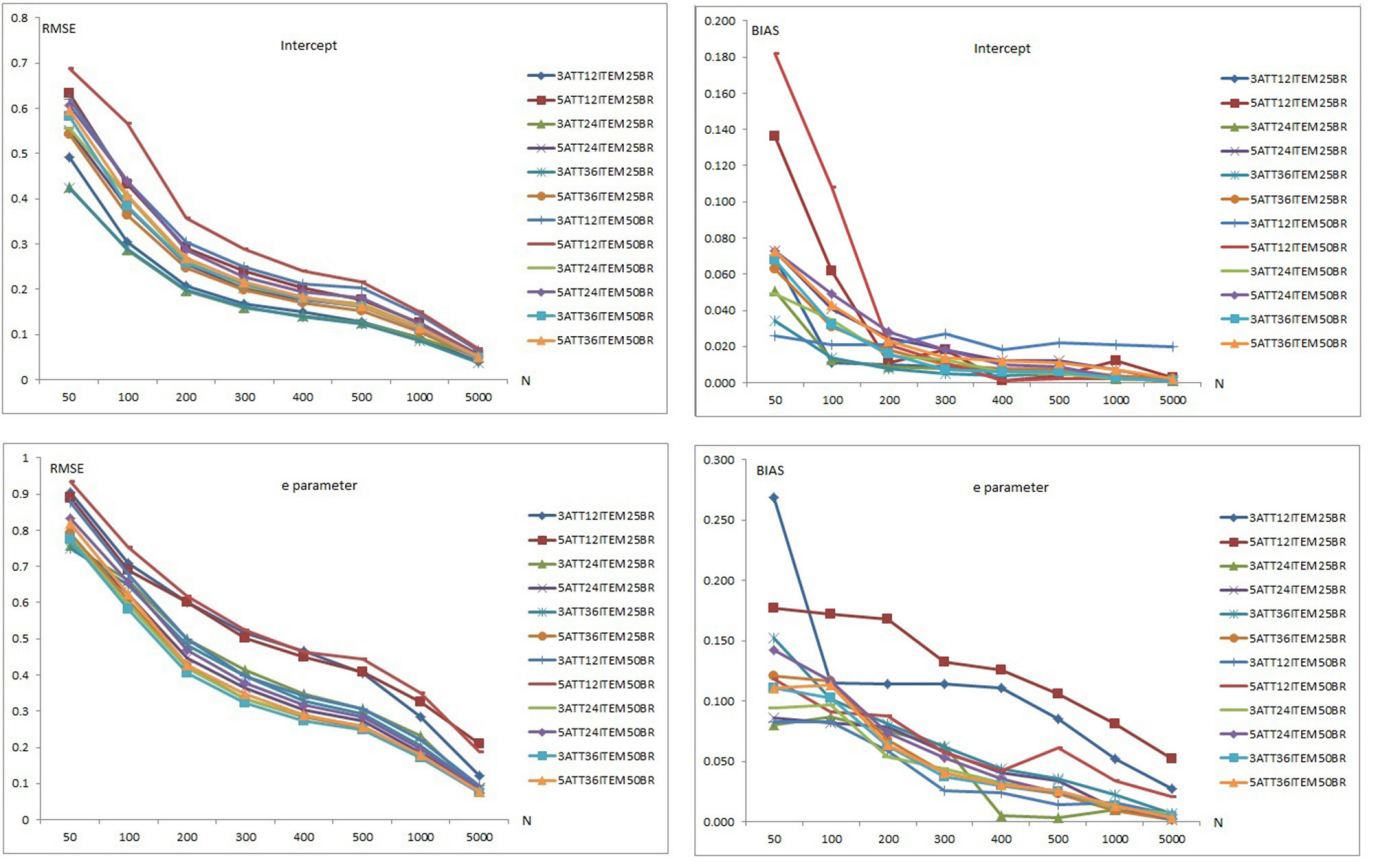
Mean RMSE and bias plots for the DINO Model.

**Figure 4:**
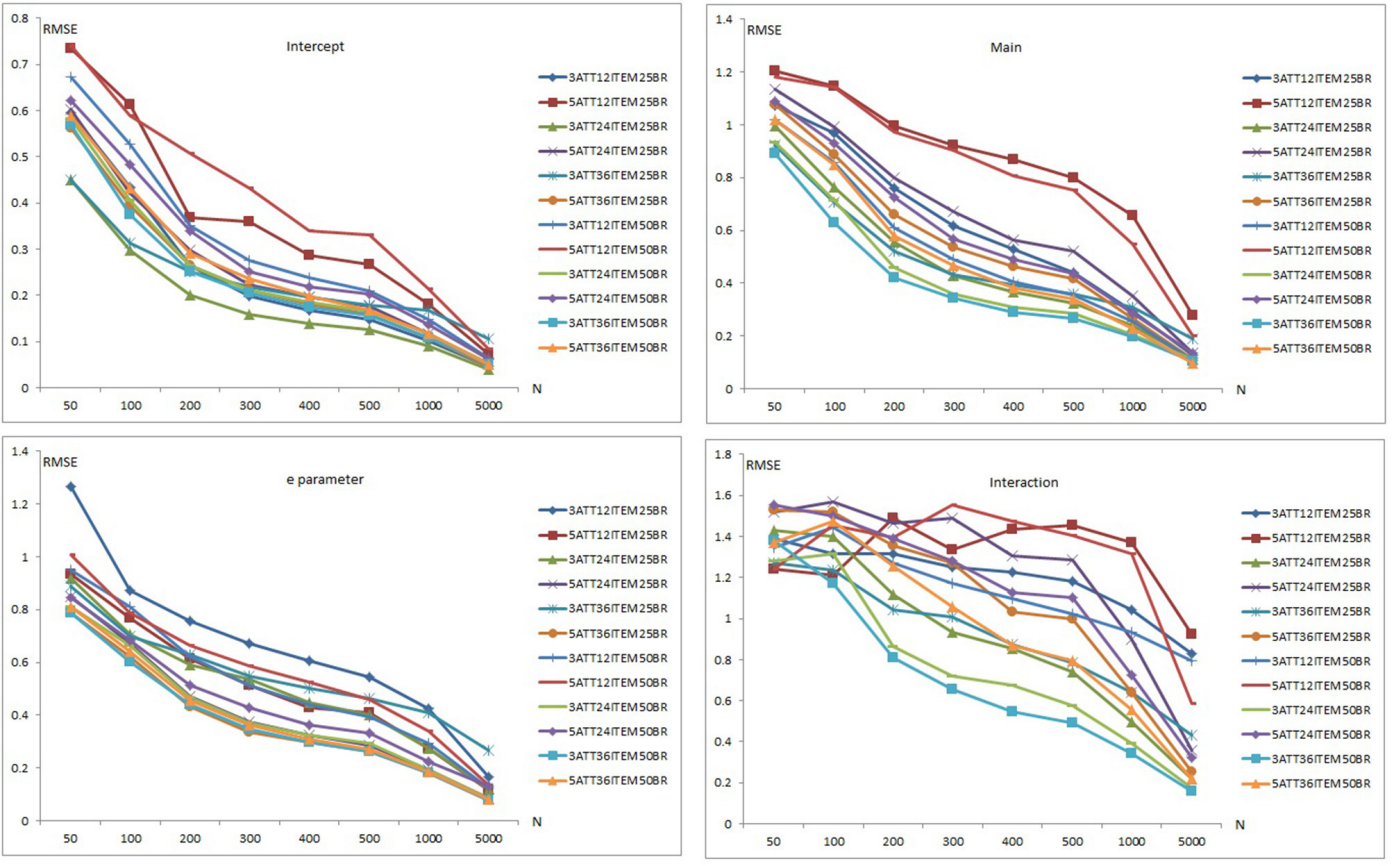
Mean RMSE plots for the LCDMREDUCED Model.

**Figure 5:**
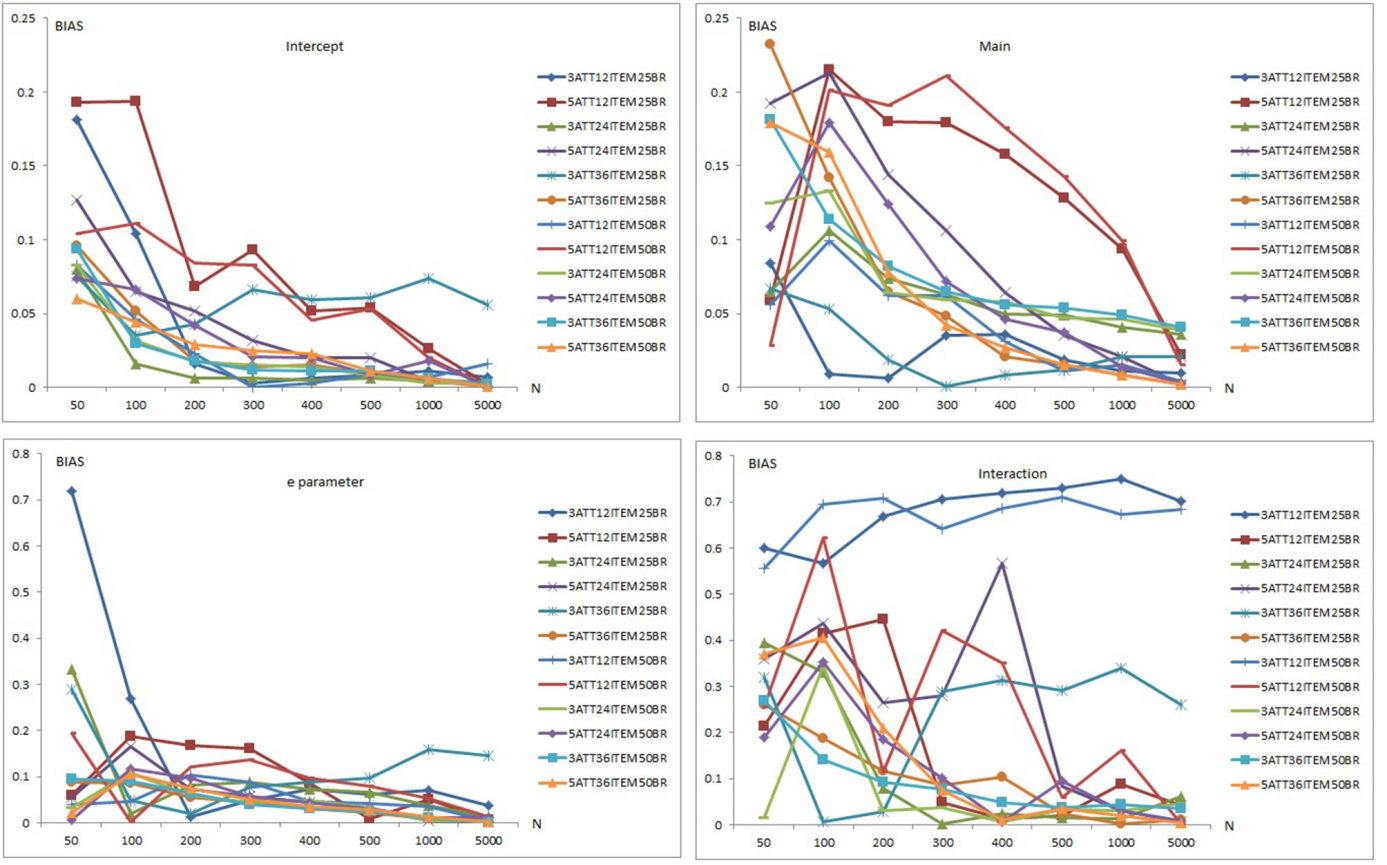
Mean bias plots for the LCDMREDUCED Model.

Results of item recovery and classification accuracy were obtained for the 384 conditions in the study. Mean RMSE and mean bias values were computed over 100 replications. The percentage of classification accuracy was also calculated for each condition. Mean RMSE and bias values are presented in Supplementary Tables 1–12. Figures 1–**5** summarize the mean RMSE and absolute mean bias results for each fitted model. Separate plots are provided for intercept, main, *e* parameter, and interaction effects in each figure. Each plot displays 12 labeled lines representing 12 different conditions for the 2 attributes, 3 test lengths, and 2 base rates. For instance, 3ATT12ITEM25BR label represents a condition with 3 attributes, 12 items, and 0.25 base rate of mastery.

### Item Parameter Recovery Results

#### Recovery of the C-RUM

Figure 1 presents the item parameter recovery results for the C-RUM when model-data fit holds. As can be seen in Figure 1, mean RMSE and bias values for the intercept and main parameters of the C-RUM appear to decrease as the sample size increases. Mean RMSE values for the intercept parameter ranged from 0.040 to 0.759 (see Supplementary Tables 1–3). Mean bias values for the intercept parameter were between 0.001 and 0.259.

Bias and RMSE values for the intercept parameter in 12-item conditions was higher than in the 24-item conditions, which was than the 36-item conditions. The 5-attribute conditions yielded higher RMSE and bias values than the 3-attribute conditions, and the 0.50 base rate conditions yielded higher RMSE and bias values than 0.25 base rate conditions. Overall, the highest mean RMSE values were for the 5-attribute × 12-item × 0.50 base rate conditions and the lowest mean RMSE and bias values were for the 3-attribute 36-item × 0.25 base rate conditions. Similarly, the highest mean bias values were obtained in the 5-attribute × 12-item for both 0.25 and 0.50 base rate conditions. The lowest mean bias values were in the 3-attribute × 36-item × 0.25 base rate conditions.

The RMSE and bias plots for intercept indicate that sample size, test length, and base rate of mastery had effects on mean RMSE and bias for the intercept parameter. It appears that the number of respondents, test lengths, number of attributes, and mastery base rates had an impact on the recovery of intercept parameter. As the number of respondents increased and test length, number of attributes, and base rate decreased, the recovery of the intercept parameter appeared to increase. Only conditions with 5,000 respondents produced RMSE values <0.10. Some of the 1,000-respondent conditions also yielded RMSE values <0.10. Mean RMSE values for the conditions <200 exceeded 0.20. The conditions with 12 items produced highest bias values. A sharp decline was observed with other conditions after 200 respondents.

In the lower panels in Figure 1, the item recovery values are plotted for the main effect of the C-RUM. As can be seen, both RMSE and bias values decreased as the number of respondents increased. This pattern is clearer for the RMSE plot (see the left lower panel) than of the bias plot (see the right lower panel). Mean RMSE values for the main effect ranged from 0.080 to 1.210 (see Supplementary Tables 1–3). Mean bias values ranged between 0.001 and 0.297. Item parameter recovery values for the main effect parameter in the 12-item conditions was higher than in the 24-item conditions and both were higher than in the 36-item conditions. The 5-attribute conditions yielded higher RMSE and bias values than the 3-attribute conditions. The pattern is clearer for RMSE as some of the conditions showed reversals for mean bias values. The 0.50 base rate conditions yielded higher RMSE values than the 0.25 base rate conditions. Except for the 36-item conditions, the 0.50 base rate conditions yielded higher bias values than the 0.25 base rate conditions.

Overall, the highest mean RMSE values were obtained with the 5-attribute × 12-item × 0.25 base rate conditions while the lowest mean RMSE values were observed with the 3-attribute × 36-item × 0.50 base rate conditions. Similarly, the highest mean bias values were obtained with 5-attribute × 12-item for both 0.25 and 0.50 base rate conditions. The lowest mean bias values were observed with 3-attribute × 36-item × 0.50 base rate conditions. The RMSE and bias plots for the intercept indicate that sample size, test length, and base rate of mastery had effects on mean RMSE and bias for the main effect parameter. The number of respondents, test length, number of attributes, and base rate of mastery also had effects on the recovery of the intercept parameter. As the number of respondents and base rate increased and test length and number of attributes decreased, the recovery of the main effect parameter increased. Except for the 12-item conditions, only conditions with 5,000 respondents produced RMSE values <0.10. Mean RMSE values for the main effect under conditions with <1,000 exceeded 0.20. The conditions with 12 items produced the highest bias values. Bias values <0.10 were more likely under conditions with more than 200 respondents. Overall, the recovery of intercept parameter was found to be better than that of the main effect parameter for the C-RUM.

#### Recovery of the DINA Model

Figure 2 shows item recovery results for the DINA model when model-data fit holds. As can be seen in Figure 2, mean RMSE and bias values for intercept and *e* parameter of the DINA model decreased as sample size increased. Mean RMSE values for the intercept parameter ranged from 0.037 to 0.628 (see Supplementary Tables 4–6). Mean bias values for the intercept parameter ranged from 0.001 to 0.218. When the data generating model was the DINA, recovery values for the intercept parameter in the 12-item conditions were higher than in the 24-item conditions and both were higher than in the 36-item conditions. The 5-attribute conditions yielded higher RMSE and bias values than the 3-attribute conditions.

The 0.50 base rate conditions yielded higher RMSE values than the 0.25 base rate conditions for the intercept parameter. The 0.50 base rate conditions produced lower RMSE values than the 0.25 base rate conditions for the 3-attribute conditions. However, the 0.25 base rate conditions produced lower values than for the 0.50 base rate conditions under most of the 5-attribute conditions.

Overall, the highest mean RMSE values were obtained with 5-attribute × 12-item × 0.50 base rate conditions while the lowest mean RMSE values were observed with 3-attribute and 0.25 base rate conditions with 24 and 36 items. Similarly, the highest mean bias values were obtained with 5-attribute × 12-item conditions for both the 0.25 and 0.50 base rate conditions. The lowest mean bias values were observed with the 3 attribute × 0.25 base rate conditions with 24 and 36 items. It appears that the number of respondent, test length, number of attributes, and base rate of mastery had an impact on the recovery of the intercept parameter. As the number of respondents and test length increased, and the number of attributes and base rate decreased, the recovery of the intercept parameter appeared to increase. Only conditions with 5,000 respondents with 24 and 36 items produced RMSE values <0.10. Mean RMSE values, however, were found to be higher than 0.10 for 12-item conditions even with 5,000 respondents. For 24- and 36-item conditions, mean RMSE values exceeded 0.20 with sample sizes <200. This was not the case with the 12-item conditions as conditions with 12 items also produced the highest bias values. Mean bias values for the intercept parameter were found to be between 0.001 and 0.10 under most of the conditions except for two conditions: 5-attribute × 12-item × 0.25 base rate and 5-attribute × 12-item × 0.50 base rate.

As can be seen in the lower part of Figure 2, similar patterns were observed for recovery of the *e* parameter. However, both RMSE and bias values of the *e* parameter were higher than for the intercept parameter.

Mean RMSE values for the *e* parameter ranged from 0.080 to 1.067 (see Supplementary Tables 4–6). Mean bias values for the *e* parameter were between 0.001 and 0.592. When the data generating model was the DINA model, item recovery values for the *e* parameter in the 12-item conditions was higher than in the 24-item conditions and both were higher than in the 36-item conditions. The 3-attribute conditions yielded higher RMSE values than the 5-attribute conditions for the 0.25 base conditions. However, the 5-attribute conditions yielded higher RMSE values than the 3-attribute conditions for the 0.50 base rate of mastery. Mean bias results for the *e* parameter did not show any clear pattern with respect to the number of attributes and base rate.

Overall, the highest mean RMSE values for *e* parameter were obtained with the 3-attribute × 12-item × 0.25 base rate conditions while the lowest mean RMSE values were observed for the 36-item × 0.50 base rate conditions with 3 and 5 attributes. Similarly, the highest mean bias values for the *e* parameter were obtained with the 5-attribute × 12-item conditions with both the 0.25 and 0.50 base rates. The lowest mean bias values were observed with 3-attribute × 0.25 base rate conditions with both 24 and 36 items. It appears that the number of respondents, test length, number of attributes, and mastery base rates had an impact on the recovery of the intercept parameter. As the number of respondents and test length increased, the recovery of the *e* parameter appeared to increase. The effect of number of attributes and base rates appeared to be less clear. Only the 24- and 36-item conditions with 5 attributes produced RMSE values <0.10 when the sample size was 5,000. As can be seen in Supplementary Tables 4–6, a few RMSE values <0.20 were observed, even with 1,000 respondents. Overall, the intercept parameter of the DINA model appeared to be recovered better than the *e* parameter based on mean RMSE and bias values.

#### Recovery of the DINO Model

Item recovery results for the DINO model (see Figure 3), indicate that mean RMSE and bias values for intercept and *e* parameter of DINO model appear to decrease as the number of respondents increases when model-data fit holds. Mean RMSE values for intercept parameter ranged from 0.038 to 0.688 (see Supplementary Tables 7–9). Mean bias values for the intercept parameter ranged between 0.001 and 0.182. Item recovery values for the intercept parameter in the 12-item conditions was higher than in the 24-item conditions and both were higher than in the 36-item conditions. The 5-attribute conditions yielded higher RMSE and bias values than the 3-attribute conditions for the intercept parameter.

The 0.50 base rate conditions yielded higher RMSE values than 0.25 base rate conditions for the intercept parameter. The 0.25 base rate conditions also yielded lower values than the 0.50 base rate conditions under most of the 3-attribute conditions. However, the 0.25 base rate conditions produced lower values than the 0.50 base rate conditions for over half of the 5-attribute conditions.

Overall, the highest mean RMSE values for the intercept parameter were obtained for the 5-attribute × 12-item × 0.50 base rate conditions while the lowest mean RMSE values were observed with the 3-attribute × 0.25 base rate conditions for both 24 and 36 items. Similarly, the highest mean bias values were obtained with the 5-attribute × 12-item × 0.25 base rate conditions in addition to 5-attribute 12-item and 0.50 base rate under the small sample size conditions (i.e., <200). However, the highest mean bias values were obtained with the 3-attribute × 12-item × 0.50 base rate conditions for sample sizes >200. The lowest mean bias values for the intercept parameter of DINO model were observed with the 3-attribute × 0.25 base rate conditions for both 24 and 36 items. It appears that the number of respondents, test length, number of attributes, and mastery base rates had an impact on recovery of the intercept parameter. As the number of respondents and test length increased, and number of attributes and base rate decreased, the recovery of intercept parameter appeared to improve. Conditions with 5,000 simulated respondents produced RMSE values <0.100. The conditions with 12 items also produced the highest bias values for the intercept parameter. Mean bias values for the intercept parameter were between 0.001 and 0.100 except for two conditions: The 5 attribute × 12 item × 0.25 base rate and the 5 attribute × 12 item × 0.50 base rate conditions.

As can be seen in the lower panels of Figure 3, similar patterns were observed with the recovery of the *e* parameter, although both RMSE and bias values were higher than for the intercept parameter. Mean RMSE values for the *e* parameter ranged from 0.076 to 0.934 (see Supplementary Tables 7–9). Mean bias values for the *e* parameter ranged between 0.002 and 0.269. Recovery values for the *e* parameter in the 12-item conditions were higher than in the 24-item conditions and both were higher than in the 36-item conditions. The 3-attribute conditions yielded higher RMSE values than 5-attribute conditions for the 0.25 base conditions. However, the 5-attribute conditions yielded higher RMSE values than 3-attribute conditions for the 0.50 base rate conditions. For both 0.25 and 0.50 base rate conditions, mean bias values of the *e* parameter for the 5-attribute conditions were higher than for the 3-attribute conditions.

Overall, the highest mean RMSE values for the *e* parameter were with 5-attribute × 12-item × 0.50 base rate conditions while the lowest mean RMSE values were for the 3-attribute × 36-item × 0.50 base rate conditions. Similarly, the highest mean bias values for the *e* parameter were obtained with the 12-item × 0.25 base rate conditions with both 3 and 5 attributes while the lowest mean bias values were observed with 3-attribute × 12-item × 0.50 base rate conditions.

The number of respondents, test length, number of attributes, and base rates of mastery appeared to affect recovery of the intercept parameter. As the number of respondents and test length increased, recovery of the *e* parameter increased. However, the effects of the number of attributes and base rates were less clear. Only the 5-attribute conditions × 5,000 respondents for the 24 and 36 items yielded RMSE values <0.10. As can be seen in Supplementary Tables 7–9, relatively few RMSE values <0.20 were observed even with 1,000 respondents. Overall, the intercept parameter of the DINO model appeared to be recovered better than the *e* parameter.

#### Recovery of the LCDMREDUCED Model

Figures 4, 5 are plots of the mean RMSE and bias results, respectively, for the LCDMREDUCED model when model-data fit holds. The mean RMSE and bias summaries are presented separately as the number of estimated parameters in the LCDMREDUCED model is higher than C-RUM, DINA, and DINO models. Separate recovery plots are provided for the intercept, main effect, *e* parameter, and interaction effects. As can be seen in Figure 4, mean RMSE values for the intercept, main, *e* parameter, and interaction effects of the LCDMREDUCED model decreased as sample size increased. Mean RMSE values for the intercept parameter ranged from 0.041 to 0.741 (see Supplementary Tables 10–12). Mean RMSE values for main effects ranged between 0.096 and 1.206. Mean RMSE values for the *e* parameter ranged from 0.078 to 1.266 (see Supplementary Tables 10–12). Mean RMSE values for the interaction effects ranged between 0.158 and 1.569.

When the data generating model was the LCDMREDUCED, mean RMSE values for the intercept, main effects, *e* parameter, and interaction effects in the 12-item conditions were higher than in the 24- and 36-item conditions. However, the 36-item conditions yielded smaller RMSE values than the 24-item conditions under more than half of the conditions. The 0.25 base rate conditions yielded lower RMSE values than the 0.50 base rate conditions except for the main effect parameters under the 12-item conditions. The base rate did not show any clear pattern of effects for the 24-item and 36-item conditions. Except for the 0.25 base rate for the *e* parameter, the 3-attribute conditions produced lower mean RMSE values than the 5-attribute conditions for intercept, main, *e* parameter, and interaction effects.

Overall, the highest mean RMSE values were obtained with 5-attribute × 12-item conditions for both 0.25 and 0.50 base rate conditions for the intercept, main effects and interaction parameters. The highest RMSE for the *e* parameter was observed with the 3-attribute × 12-item × 0.25 base rate conditions. The lowest mean RMSE values were observed with the 3-attribute × 36-item × 0.50 base rate conditions for the main effects, *e* parameter, and interaction effects. The 3-attribute × 24-item × 0.25 base rate conditions yielded the lowest RMSE values for the intercept parameter.

Separate bias plots are presented in Figure 5 for the intercept, main effects, *e* parameter, and interaction effects. As can be seen in Figure 5, mean bias values for intercept, main effect, *e* parameter, and interaction effects of the LCDMREDUCED model appeared to decrease as the number of respondents increased. Mean bias plots of the intercept and *e* parameter showed clearer patterns than those of the main and interaction effects. Mean bias values for the intercept parameter ranged from 0.001 to 0.194 (see Supplementary Tables 10–12). Mean bias values for main effects ranged between 0.001 and 0.232. Mean bias values for the *e* parameter ranged from 0.002 to 0.718 (see Supplementary Tables 10–12). Mean bias values for interaction effects were between 0.002 and 0.750.

When the data generating model was the LCDMREDUCED, mean bias values for the intercept, main, *e* parameter, and interaction effects in the 12-item conditions were higher than in the 24- and 36-item conditions. The 36-item conditions yielded smaller bias values than the 24-item conditions for more than half of these conditions. Mean bias values showed less clear patterns with respect to base rate and number of attributes.

Overall, the highest mean bias values were obtained with the 5-attribute × 12-item conditions under both the 0.25 and 0.50 base rate conditions for intercept, main effects and *e* parameters. In addition, the 3-attribute × 36-item × 0.25 base rate conditions yielded higher RMSE values for sample sizes >400. The highest mean bias values for the interaction effect were for the 3-attribute × 12-item conditions under both 0.25 and 0.50 base rate conditions. There was no clear pattern of lowest mean bias values, however, for the intercept, main, *e* parameter, and interaction effects.

It appears that the number of respondent, test length, number of attributes, and base rate of mastery had an impact on the recovery of LCDMREDUCED parameters. As the number of respondents and test length increased, the recovery of LCDMREDUCED parameter also improved.

Only the intercept parameter conditions produced RMSE values <0.10 when the sample size was 5,000. Main effect RMSE results were also very close to 0.10 when the sample size was 5,000. As can be seen in Supplementary Tables 10–12, RMSE values tended to be higher than 0.20 even with 1,000 respondents. Overall, the intercept parameter of the LCDMREDUCED model appeared to be recovered better than the other parameter based on mean RMSE and bias values, and recovery of the interaction effect appeared to be less accurate than for the other parameters.

### A Linear Model Analysis of Item Recovery Statistics

Mean RMSE and bias results were summarized using a linear model. Effects of each of the different conditions for each of the RMSE and bias values were assessed using a factorial ANOVA. Table 5 presents partial eta-squared values for each of the main effects and the two-way interactions. As can be seen in Table 5, sample size (*N*) was the most influential factor on RMSE and bias for each item parameter except for interaction bias. Test length (*k*) was the second most influential factor on RMSE and bias values. Base rate of mastery was the least influential factor on RMSE and bias calculated for intercept and the interaction parameters. Model type was the least influential factor on RMSE and bias calculated for the main effect parameters. Number of attributes was the least influential factor on RMSE and bias for the *e* parameter. Effects of two-way interactions between simulated factors appeared to be less than for main effects (see Table 5). Most of the main and interaction effects were found to have significant effects on RMSE and bias values.

**Table 5:** Partial eta-squared values for manipulated variables in simulation.

	**RMSE**				**Bias**			
	**Intercept**	**Main**	**e parameter**	**Interaction**	**Intercept**	**Main**	**e parameter**	**Interaction**
*N*	0.976^*^	0.983^*^	0.985^*^	0.960^*^	0.469^*^	0.674^*^	0.356^*^	0.407
Att	0.540^*^	0.810^*^	0.167^*^	0.788^*^	0.119^*^	0.349^*^	0.001	0.406
*k*	0.777^*^	0.903^*^	0.800^*^	0.835^*^	0.236^*^	0.197^*^	0.113^*^	0.739
BR	0.420^*^	0.460^*^	0.339^*^	0.452^*^	0.007	0.004	0.090^*^	0.038
Model	0.633^*^	0.446^*^	0.404^*^	-	0.049^*^	0.001	0.018	-
*N*×Att	0.127^*^	0.314^*^	0.019	0.552^*^	0.094^*^	0.247^*^	0.236^*^	0.300
*N*×*k*	0.274^*^	0.543^*^	0.217^*^	0.772^*^	0.077^*^	0.492^*^	0.037	0.222
*N*×BR	0.137^*^	0.077	0.039	0.206	0.055^*^	0.022	0.164^*^	0.107
*N*×Model	0.328^*^	0.158^*^	0.080	-	0.053	0.014	0.054	-
Att×*k*	0.198^*^	0.334^*^	0.148^*^	0.354^*^	0.059^*^	0.092^*^	0.010	0.673
Att×BR	0.074^*^	0.005	0.575^*^	0.060	0.000	0.040^*^	0.042^*^	0.036
Att×Model	0.025	0.121^*^	0.247^*^	-	0.040^*^	0.003	0.021	-
*k*×BR	0.048^*^	0.001	0.016	0.187^*^	0.015	0.037	0.025	0.091
*k*×Model	0.407^*^	0.224^*^	0.047^*^	-	0.140^*^	0.045^*^	0.012	-
BR×Model	0.059^*^	0.001	0.070^*^	-	0.034^*^	0.014	0.008	-

*N, sample size; Att, number of attributes; k, test length; BR, base rate of mastery; Model, model type*;

* *p < 0.01*.

### Classification Accuracy Results

**Figure 6:**
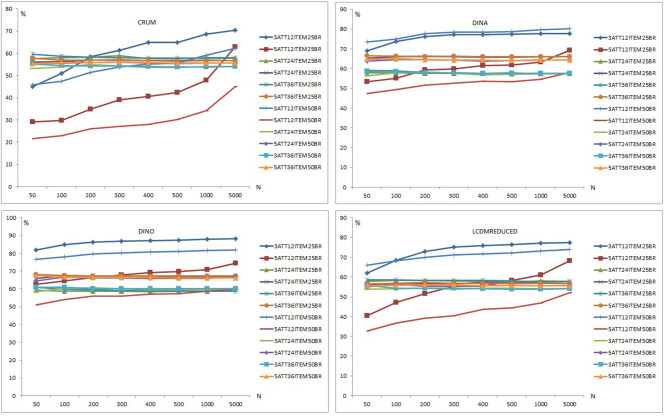
Classification accuracy plots for the C-RUM, DINA, DINO, and LCDMREDUCED Models.

Figure 6 presents the classification accuracy results for C-RUM, DINA, DINO, and LCDMREDUCED models when model-data fit holds. Classification accuracy percentages appear to increase for the 12-item conditions (i.e., conditions 3ATT12ITEM25BR, 3ATT12ITEM50BR, 5ATT12ITEM25BR, 5ATT12ITEM50BR) as the number of respondents increases. The classification accuracy percentages appear to change only slightly for the 24- and 36-item conditions across the different sample sizes.

For the C-RUM, classification accuracy percentages ranged from 21.640 to 70.344 (see Supplementary Table 13). When the data generating model was the C-RUM, classification accuracy results were very close for the 24- and 36-item conditions. Neither the number of attributes nor the base rate, however, appeared to have a significant effect on the classification accuracy for the 24- and 36-item conditions. The sample size increase also appears to have slight effect on the classification accuracy for the 24- and 36-item conditions. C-RUM results varied for the 12-item conditions. The 5-attribute × 12-item × 0.50 base rate conditions had the lowest percentages (see Figure 6). The 3-attribute × 12-item × 0.25 base rate conditions produced the highest percentages for the C-RUM for sample sizes >200.

For the DINA model, classification accuracy percentages ranged from 47.480 to 80.210 (see Supplementary Table 13). For the DINA generating model, classification accuracy results for the 24- and 36- item conditions were very close. The 5-attribute conditions had higher percentages than the 3-attribute conditions for the 24- and 36-item conditions. The increase in sample size appeared to have a slight effect on the classification accuracy for the 24- and 36-item conditions. However, DINA model results varied for the 12-item conditions. The 5-attribute × 12-item × 0.50 base rate conditions produced the lowest percentages for the DINA model. The 3-attribute × 12-item conditions for both 0.25 and 0.50 base rates produced the highest percentages. Results for the 12-item conditions appear to be stable for sample sizes of 200 or more.

For the DINO model, classification accuracy percentages ranged from 51.00 to 88.27 (see Supplementary Table 13). When the data generating model was the DINO model, classification accuracy results were similar for the 24- and 36-item conditions. The 5-attribute conditions had higher percentages than the 3-attribute conditions for both 24- and 36-item conditions. The sample size increase appears to have a slight effect on the classification accuracy under the 24- and 36-item conditions. The 5-attribute × 12-item × 0.50 base rate conditions had the lowest percentages for the DINO model. The 3-attribute × 12-item × 0.25 base rate conditions produced the highest percentages. DINO model results for 12 items appeared to be more stable for samples of 200 or more.

For the LCDMREDUCED, classification accuracy percentages ranged from 32.820 to 77.456 (see Supplementary Table 13). When the data generating model was the LCDMREDUCED, classification accuracy results were similar between the 24- and 36-item conditions. The 5-attribute conditions had higher percentages than the 3-attribute conditions for both 24- and 36-item conditions. The sample size increase appears to have a slight effect on the classification accuracy under the 24- and 36-item conditions. LCDMREDUCED results were variable for the 12-item conditions. The 5-attribute × 12-item × 0.50 base rate conditions had the lowest percentages for the LCDMREDUCED, and the 3-attribute × 12-item × 0.25 base rate conditions had the highest percentages for LCDMREDUCED model.

The number of respondents, number of attributes, and mastery base rate had an impact on the classification accuracy of DCMs in the 12-items conditions. When all models were compared, the highest classification was observed with the DINO model (the mean across all conditions = 66.35). The models with the next highest classification percentages after the DINO model were the DINA, LCDMREDUCED and C-RUM models, respectively. The average classification percentages across all conditions were 63.16, 57.54, and 52.82 for the DINA, LCDMREDUCED, and C-RUM models, respectively.

## Discussion

The present simulation study was designed to investigate the effects of sample size on item parameter recovery and classification accuracy of four DCMs, the C-RUM, DINA, DINO, and LCDMREDUCED. Effects of additional factors including test length, number of attributes, and base rate of mastery were also examined. Bias and RMSE values were computed between true generating parameters and estimated parameters. Effects of simulated factors on attribute assignment were also evaluated using the percentage of classification accuracy.

The present study differed from previous studies (Rojas et al., 2012; Başokcu, 2014) in several respects. Although previous simulations on DCMs showed that classification accuracy and item recovery can be poor with small sample sizes, they tended to focus on a limited number of sample size conditions, making results somewhat difficult to generalize to other practical testing conditions. This study extended the sample size conditions from 50 to 5,000. Results showed that sample size appears to have an impact on recovery of DCM model parameters. Larger sample sizes showed better item parameter recovery. The effect of sample size on item parameter recovery is consistent with previous research (Rojas et al., 2012; Başokcu, 2014). Conditions with sample sizes <200 showed poor results. In general, it appears that sample sizes should be at least 500 for the four DCMs considered in this study in order to obtain precise estimates. This is consistent with previous research in which a sample size of 500 was considered to be a small sample size for DCMs (Bradshaw and Madison, 2016; Madison and Bradshaw, 2018), although small RMSE and bias values were difficult to obtain with samples of 1,000 respondents under some conditions. The results of this simulation study showed that a sample size as small as *N* = 1,000 would be sufficient to adequately recover all model parameters, under all the given conditions, adequately for the DINA, DINO and C-RUM models. However, the LCDMREDUCED model does appear to require larger sample sizes for some model parameters such as the interaction effect.

Another important finding from this study is that increase in test length did result in more precise estimates of item parameters. The average RMSE and bias values decreased as test length increased from 12 to 36 items. This finding is consistent with previous research indicating that the CDM framework requires assessments that are at least of moderate test lengths of 15 or 20 items (de la Torre, 2009).

Another important finding obtained is the effect of number of attributes on the recovery of item parameter estimates. The recovery of item parameters worsened when the number of attributes increased from three to five. This is important as the most of the studies in DCM literature use more than three attributes. For example, Sessoms and Henson (2018) has conducted a literature review on the applications of DCMs and found that number of attributes estimated varied from four to 23. The average number of attributes estimated was 8 and almost half of the application studies modeled 8 or more attributes. As shown in this simulation study, higher numbers of attributes clearly required larger sample sizes. Results of this study, in other words, suggest that tests with large numbers of attributes also need larger sample sizes to accurately estimate model parameters.

Another important point to be noted is the distribution of attributes over items. In this study, the distribution of attributes over items was not equal for the five-attribute case. This does have an effect on estimation. For instance, Madison and Bradshaw (2015) compared performance of the LCDM using various Q-matrix designs and found that classification accuracy varied markedly for different Q-matrix designs. For a given number of items an attribute is measured, classification accuracy increased as the number of items measuring the attribute in isolation increased. In contrast, classification accuracy suffered most when a pair of attributes was measured. In this study, the same numbers of attributes over items were used in the LCDMREDUCED model for the four models that were estimated (C-RUM, DINA, DINO, and LCDM). It is important to note that operational tests are constructed to meet the requirements of test blueprints so may not have this type of regular pattern. As is the case for any simulation study, the generalizability of the results of this study is necessarily limited to conditions manipulated in this study. It would be helpful, in this regard, to study the effects of different patterns of attributes in future research. Among the limitations of this study are the lacks of consideration of a general model (e.g., LCDM) and of the number of attributes larger than five.

This simulation study also showed that mastery base rate had a varying effect on item parameter recovery. This effect varied for item parameter types (i.e., intercept, main, e parameter, and interaction effects) and model type. It appears that intercept and *e* parameters were better recovered than main and interaction parameters. Consistent with previous research (Kunina-Habenicht et al., 2012; Bradshaw and Madison, 2016), the recovery of the interaction terms was lower than for the intercepts, main effects, and *e* parameters.

Parameters of DINA and DINO models were more likely to be recovered well than C-RUM and LCDMREDUCED models when model-data fit holds. This was also the case for classification accuracy. The DINO model had better fit with small sample sizes than the other three DCMs. This result is consistent with previous research (Roussos et al., 2007; de la Torre, 2011). Previous simulation studies generated data sets under the assumption of a common underlying model for the whole test. The simulation in the present study also considered different underlying model for each model (i.e., the LCDMREDUCED model). The LCDMREDUCED model consisted of four different model structures including the C-RUM, DINA, DINO, and full LCDM. The item parameter recovery and classification accuracy of the LCDMREDUCED model was worse than for the other DCMs.

Patterns of bias and RMSE values computed between true (i.e., generating) parameters and estimated parameters were consistent for almost all conditions. However, some irregularities were observed in which reversals occurred in the results for bias. Previous research has also reported this finding in which different patterns occurred for bias compared to RMSE (Harwell, 2018). As Harwell has noted, it may be that average bias is masking important patterns in recovery accuracy compared to RMSE.

Consistent with previous research (de la Torre et al., 2010; Rojas et al., 2012), the sample size did not result in any change in classification accuracy percentages for the 24 and 36 item conditions. Higher attribute assignments, however, were observed with larger sample sizes in the 12-item conditions. Overall, the classification accuracy rates were below 90% even for the 5,000 sample size. Consistent with previous research (de la Torre et al., 2010; Kunina-Habenicht et al., 2012; Lei and Li, 2016), results from the present study showed that sample size explained only a small proportion of the variance in classification accuracy. Higher attribute assignments were observed with the 12-item conditions, compared to the 24- and 36-item conditions. This was expected as the longer test lengths provide more information on which the classifications can be based.

Classifications with the DINO and DINA models were more accurate than the C-RUM and LCDMREDUCED models. The simulation study results showed that the DINO and DINA models performed better in this regard for small samples than other two DCMs.

Several practical suggestions may be made from this study for researchers or practitioners who seek to design diagnostic tests from a DCM framework. Results of this study showed that simpler models were recovered better than more complex models. Thus, before drawing any conclusion based on a specific DCM, one might alternatively specify other appropriate DCMs, which can capture potential relationships among the attributes. Another finding of this study was the increasing accuracy of the recovery of the item parameters as sample sizes increased. For instance, to ensure model identifiability and consistent estimation, it is necessary to collect sufficient data (i.e., typically samples of 1,000 or more) that satisfy identifiability, when designing the diagnostic tests. It was also shown that longer tests produced more precise and consistent estimates. Results of this study showed varying effects of mastery base rate on item parameter recovery. It would be useful to explore this issue in future research.

The probability of making a correct classification and accurately recovering item parameters depends at least in part on the fit of the model to the data. In this study, model-data fit was assumed for each condition, as the generating and estimated models were the same. It is difficult to know in practice, however, whether the selected DCM is the best fitting model to real test data. As Ma (2020) has noted, the usefulness of DCMs depends on whether they can adequately fit the data. It is for this reason that fit indices play an important role in selecting the best fitting DCM. Several studies have been conducted to examine the performances of absolute fit (Hu et al., 2016) and relative fit indices (de la Torre and Douglas, 2008; Hu et al., 2016; Sen and Bradshaw, 2017) in the DCM framework. These studies can help guide practitioners to choose appropriate model fit indices to estimate model fit under various conditions.

Results of this study suggest that the precision of the parameter estimates and classification accuracy are a function not only of the sample size but also of test length, number of attributes, base rate of mastery, and model type. Selection of the appropriate DCMs needs to be guided in part by sample size.

## Data Availability Statement

The raw data supporting the conclusions of this article will be made available by the authors, without undue reservation.

## Author Contributions

SS and AC contributed equally to the data analyses and reporting parts. Both authors contributed to the article and approved the submitted version.

## Conflict of Interest

The authors declare that the research was conducted in the absence of any commercial or financial relationships that could be construed as a potential conflict of interest.

## References

[B1] Akaike H. (1974). A new look at the statistical model identification. IEEE Trans. Autom. Control 19, 716–723. 10.1109/TAC.1974.1100705

[B2] Akbay L. (2016). Relative efficiency of the nonparametric approach on attribute classification for small sample cases. J. Eur. Educ. 6, 19–35. 10.18656/jee.58934

[B3] Başokcu T. O. (2014). Classification accuracy effects of Q-matrix validation and sample size in DINA and G-DINA models. J. Educ. Pract. 5, 220–230. 10.7176/JEP

[B4] Bozdogan H. (1987). Model selection and akaike's information criterion (AIC): the general theory and its analytical extensions. Psychometrika 52, 345–370. 10.1007/BF02294361

[B5] Bradshaw L. P. Madison M. J. (2016). Invariance properties for general diagnostic classification models. Int. J. Testing 16, 99–118. 10.1080/15305058.2015.1107076

[B6] Chen H. Chen J. (2016). Retrofitting non-cognitive-diagnostic reading assessment under the generalized DINA model framework. Lang. Assess. Q. 13, 218–230. 10.1080/15434303.2016.1210610

[B7] Chiu C. Y. Douglas J. (2013). A nonparametric approach to cognitive diagnosis by proximity to ideal response patterns. J. Classif. 30, 225–250. 10.1007/s00357-013-9132-9

[B8] Choi H. J. Templin J. L. Cohen A. S. Atwood C. H. (2010). The impact of model misspecification on estimation accuracy in diagnostic classification models, in Paper Presented at the Meeting of the National Council on Measurement in Education (Denver, CO).

[B9] Cui Y. Gierl M. J. Chang H. H. (2012). Estimating classification consistency and accuracy for cognitive diagnostic assessment. J. Educ. Meas. 49, 19–38. 10.1111/j.1745-3984.2011.00158.x

[B10] de la Torre J. (2009). A cognitive diagnosis model for cognitively based multiple-choice options. Appl. Psychol. Meas. 33, 163–183. 10.1177/014662160832052331235984

[B11] de la Torre J. (2011). The generalized DINA model framework. Psychometrika 76, 179–199. 10.1007/s11336-011-9207-7

[B12] de la Torre J. Chiu C. Y. (2016). A general method of empirical Q-matrix validation. Psychometrika 81, 253–273. 10.1007/s11336-015-9467-8 25943366

[B13] de la Torre J. Douglas J. A. (2004). A higher-order latent trait model for cognitive diagnosis. Psychometrika 69, 333–353. 10.1007/BF02295640

[B14] de la Torre J. Douglas J. A. (2008). Model evaluation and multiple strategies in cognitive diagnosis: an analysis of fraction subtraction data. Psychometrika 73, 595–624. 10.1007/s11336-008-9063-2

[B15] de la Torre J. Hong Y. Deng W. (2010). Factors affecting the item parameter estimation and classification accuracy of the DINA model. J. Educ. Meas. 47, 227–249. 10.1111/j.1745-3984.2010.00110.x

[B16] de la Torre J. Lee Y. S. (2010). A note on the invariance of the DINA model parameters. J. Educ. Meas. 47, 115–127. 10.1111/j.1745-3984.2009.00102.x

[B17] Galeshi R. Skaggs G. (2016). Parameter recovery of a cognitive diagnostic model: evidence from a simulation study. Int. J. Quant. Res. Educ. 3, 223–241. 10.1504/IJQRE.2016.082386

[B18] Haertel E. H. (1989). Using restricted latent class models to map the skill structure of achievement items. J. Educ. Meas. 26, 333–352. 10.1111/j.1745-3984.1989.tb00336.x

[B19] Hallquist M. N. Wiley J. F. (2018). Mplus automation: an R package for facilitating large-scale latent variable analyses in Mplus. Struct. Equation Model. A Multidiscipl. J. 25, 621–638. 10.1080/10705511.2017.1402334 30083048 PMC6075832

[B20] Hartz S. M. (2002). A Bayesian framework for the unified model for assessing cognitive abilities: blending theory with practicality (Unpublished doctoral dissertation). University of Illinois at Urbana-Champaign, IL, United States.

[B21] Harwell M. (2018). A strategy for using bias and RMSE as outcomes in monte carlo studies in statistics. J. Modern Appl. Stat. Methods 17:eP2938 10.22237/jmasm/1551907966

[B22] Henson R. A. Templin J. L. Willse J. T. (2009). Defining a family of cognitive diagnosis models using log-linear models with latent variables. Psychometrika 74, 191–210. 10.1007/s11336-008-9089-5

[B23] Hu J. Miller M. D. Huggins-Manley A. C. Chen Y. H. (2016). Evaluation of model fit in cognitive diagnosis models. Int. J. Testing 16, 119–141. 10.1080/15305058.2015.1133627

[B24] Im S. Yin Y. (2009). Diagnosing skills of statistical hypothesis testing using the rule space method. Stud. Educ. Eval. 35, 193–209. 10.1016/j.stueduc.2009.12.004

[B25] Jang E. E. Dunlop M. Park G. van der Boom E. H. (2015). How do young students with different profiles of reading skill mastery, perceived ability, and goal orientation respond to holistic diagnostic feedback? Lang. Testing 32, 359–383. 10.1177/0265532215570924

[B26] Junker B. W. Sijtsma K. (2001). Cognitive assessment models with few assumptions, and connections with nonparametric item response theory. Appl. Psychol. Meas. 25, 258–272. 10.1177/01466210122032064

[B27] Kunina-Habenicht O. Rupp A. A. Wilhem O. (2012). The impact of model misspecification on parameter estimation and item-fit assessment in log-linear diagnostic classification models. J. Educ. Meas. 49, 59–81. 10.1111/j.1745-3984.2011.00160.x

[B28] Lei P. W. Li H. (2016). Performance of fit indices in choosing correct cognitive diagnostic models and Q-matrices. Appl. Psychol. Meas. 40, 405–417. 10.1177/0146621616647954 29881062 PMC5978496

[B29] Ma W. (2020). Evaluating the fit of sequential G-DINA model using limited-information measures. Appl. Psychol. Meas. 44, 167–181. 10.1177/0146621619843829 32341605 PMC7174807

[B30] Madison M. J. Bradshaw L. P. (2015). The effects of Q-matrix design on classification accuracy in the log-linear cognitive diagnosis model. Educ. Psychol. Meas. 75, 491–511. 10.1177/0013164414539162 29795830 PMC5965638

[B31] Madison M. J. Bradshaw L. P. (2018). Assessing growth in a diagnostic classification model framework. Psychometrika 83, 963–990. 10.1007/s11336-018-9638-5 30264183

[B32] Muthén L. K. Muthén B. O. (1998–2019). Mplus (Version 8.4) [Computer Software]. Los Angeles, CA: Muthén and Muthén.

[B33] Paulsen J. (2019). Examining cognitive diagnostic modeling in small sample contexts (Unpublished doctoral dissertation). Indiana University, Bloomington, IN, United States.

[B34] Rojas G. de la Torre J. Olea J. (2012). Choosing between general and specific cognitive diagnosis models when the sample size is small, in Paper Presented at the Meeting of the National Council on Measurement in Education (Vancouver).

[B35] Roussos L. A. Templin J. L. Henson R. A. (2007). Skills diagnosis using IRT-based latent class models. J. Educ. Meas. 44, 293–311. 10.1111/j.1745-3984.2007.00040.x

[B36] Rupp A. A. Templin J. Henson R. A. (2010). Diagnostic Measurement: Theory, Methods, and Applications. New York, NY: Guilford Press.

[B37] Rupp A. A. Templin J. L. (2008). Unique characteristics of diagnostic classification models: a comprehensive review of the current state-of-the-art. Measurement 6, 219–262. 10.1080/15366360802490866

[B38] Schwarz G. (1978). Estimating the dimension of a model. Ann. Stat. 6, 461–464. 10.1214/aos/1176344136

[B39] Sen S. Bradshaw L. (2017). Comparison of relative fit indices for diagnostic model selection. Appl. Psychol. Meas. 41, 422–438. 10.1177/0146621617695521 29881100 PMC5978522

[B40] Sen S. Terzi R. (2020). A comparison of software packages available for DINA model estimation. Appl. Psychol. Meas. 44, 150–164. 10.1177/0146621619843822

[B41] Sessoms J. Henson R. A. (2018). Applications of diagnostic classification models: a literature review and critical commentary. Meas. Interdiscipl. Res. Perspect. 16, 1–17. 10.1080/15366367.2018.1435104

[B42] Sünbül S. Ö. Kan A. (2016). Bilişsel tani modellerinde parametre kestirimini ve siniflama tutarliligini etkileyen faktörlerin incelenmesi. H. U. J. Educ. 31, 778–795. 10.16986/HUJE.2015014663

[B43] Tatsuoka K. K. (1983). Rule space: an approach for dealing with misconceptions based on item response theory. J. Educ. Meas. 20, 345–354. 10.1111/j.1745-3984.1983.tb00212.x

[B44] Templin J. Bradshaw L. (2014). Hierarchical diagnostic classification models: a family of models for estimating and testing attribute hierarchies. Psychometrika 79, 317–339. 10.1007/s11336-013-9362-0 24478021

[B45] Templin J. Hoffman L. (2013). Obtaining diagnostic classification model estimates using Mplus. Educ. Meas. 32, 37–50. 10.1111/emip.12010

[B46] Templin J. L. Henson R. A. (2006). Measurement of psychological disorders using cognitive diagnosis models. Psychol. Methods 11, 287–305. 10.1037/1082-989X.11.3.287 16953706

[B47] Tzou H. Yang Y. H. (2019). Improved performance of model fit indices with small sample sizes in cognitive diagnostic models. Int. J. Assess. Tools Educ. 6, 154–169. 10.21449/ijate.482005

[B48] von Davier M. (2005). A General Diagnostic Model Applied to Language Testing Data (ETS Technical Report No. RR-05–16). Princeton, NJ: Educational Testing Service. 10.1002/j.2333-8504.2005.tb01993.x 17535481

[B49] von Davier M. (2014). The Log-Linear Cognitive Diagnostic Model (LCDM) as a Special Case of the General Diagnostic Model (GDM) (RR-14-40). Princeton, NJ: Educational Testing Service 10.1002/ets2.12043

[B50] von Davier M. Yamamoto K. Shin H. J. Chen H. Khorramdel L. Weeks J. (2019). Evaluating item response theory linking and model fit for data from PISA 2000–2012. Assess. Educ. 26, 466–488. 10.1080/0969594X.2019.1586642

