# Supplementary material for: Sample Size Requirements for Applying Diagnostic Classification Models
Source: Front Psychol. 2021 Jan 25;11:621251. doi: 10.3389/fpsyg.2020.621251 (PMC7868330; doi:10.3389/fpsyg.2020.621251)
Supplement: Supplementary file 1 [file Table_1.DOCX]

Supplementary Material

# Supplementary Data

Supplementary Material

**The SAS code used to generate the .25 base rate and .70 tetrachoric correlation**

data tetra3;

input pattern nu a1 a2 a3;

datalines;

1 0.593174008 0 0 0

2 0.069095375 0 0 1

3 0.069095375 0 1 0

4 0.026990577 0 1 1

5 0.069095375 1 0 0

6 0.026990577 1 0 1

7 0.026990577 1 1 0

8 0.118568137 1 1 1;

run;

proc freq data=tetra3;

weight nu;

table a1-a3;

table a1*a2 a1*a3 a2*a3/plcorr;

run;

# Supplementary Tables

**Table S1.** Item Recovery Results for C-RUM Model with 12 Items

|  | 3 Attributes | | | | 5 Attributes | | | |
| --- | --- | --- | --- | --- | --- | --- | --- | --- |
|  | Intercept | | Main | | Intercept | | Main | |
|  | RMSE | BIAS | RMSE | BIAS | RMSE | BIAS | RMSE | BIAS |
| 25-50 | 0.739 | 0.259 | 1.134 | 0.085 | 0.721 | 0.170 | 1.210 | -0.176 |
| 25-100 | 0.584 | 0.161 | 1.083 | -0.087 | 0.640 | 0.164 | 1.191 | -0.197 |
| 25-200 | 0.455 | 0.084 | 0.951 | -0.086 | 0.495 | 0.128 | 1.074 | -0.189 |
| 25-300 | 0.361 | 0.063 | 0.872 | -0.081 | 0.431 | 0.097 | 0.984 | -0.184 |
| 25-400 | 0.248 | 0.015 | 0.777 | -0.089 | 0.388 | 0.089 | 0.929 | -0.157 |
| 25-500 | 0.256 | 0.012 | 0.708 | -0.082 | 0.363 | 0.053 | 0.900 | -0.104 |
| 25-1000 | 0.127 | -0.011 | 0.493 | -0.051 | 0.250 | 0.028 | 0.739 | -0.068 |
| 25-5000 | 0.055 | -0.010 | 0.175 | -0.022 | 0.110 | 0.007 | 0.368 | -0.015 |
| 50-50 | 0.759 | 0.192 | 1.165 | -0.160 | 0.695 | 0.086 | 1.122 | 0.168 |
| 50-100 | 0.705 | 0.125 | 1.032 | -0.133 | 0.636 | 0.076 | 1.117 | -0.109 |
| 50-200 | 0.557 | 0.101 | 0.871 | -0.138 | 0.555 | 0.063 | 1.003 | -0.092 |
| 50-300 | 0.461 | 0.085 | 0.758 | -0.108 | 0.549 | 0.093 | 0.964 | -0.089 |
| 50-400 | 0.367 | 0.035 | 0.649 | -0.071 | 0.546 | 0.115 | 0.901 | -0.088 |
| 50-500 | 0.334 | 0.028 | 0.575 | -0.067 | 0.444 | 0.055 | 0.821 | -0.087 |
| 50-1000 | 0.206 | 0.020 | 0.369 | -0.025 | 0.381 | 0.054 | 0.662 | -0.083 |
| 50-5000 | 0.075 | -0.018 | 0.133 | -0.005 | 0.123 | 0.013 | 0.233 | -0.020 |

**Table S2.** Item Recovery Results for C-RUM Model with 24 Items

|  | 3 Attributes | | | | 5 Attributes | | | |
| --- | --- | --- | --- | --- | --- | --- | --- | --- |
|  | Intercept | | Main | | Intercept | | Main | |
|  | RMSE | BIAS | RMSE | BIAS | RMSE | BIAS | RMSE | BIAS |
| 25-50 | 0.567 | 0.153 | 1.064 | 0.110 | 0.674 | 0.125 | 1.123 | -0.221 |
| 25-100 | 0.381 | 0.050 | 0.907 | -0.086 | 0.506 | 0.077 | 1.025 | -0.212 |
| 25-200 | 0.268 | 0.020 | 0.721 | -0.072 | 0.356 | 0.056 | 0.844 | -0.109 |
| 25-300 | 0.190 | 0.005 | 0.578 | -0.048 | 0.292 | 0.048 | 0.753 | -0.065 |
| 25-400 | 0.155 | -0.006 | 0.476 | -0.047 | 0.242 | 0.032 | 0.678 | -0.044 |
| 25-500 | 0.142 | 0.003 | 0.418 | -0.033 | 0.207 | 0.019 | 0.600 | -0.034 |
| 25-1000 | 0.097 | -0.001 | 0.265 | -0.024 | 0.140 | 0.014 | 0.402 | -0.017 |
| 25-5000 | 0.043 | -0.007 | 0.112 | -0.012 | 0.058 | 0.002 | 0.151 | -0.002 |
| 50-50 | 0.635 | 0.041 | 1.053 | -0.113 | 0.686 | 0.052 | 1.093 | -0.173 |
| 50-100 | 0.529 | 0.037 | 0.872 | -0.114 | 0.546 | 0.027 | 0.964 | -0.169 |
| 50-200 | 0.333 | 0.014 | 0.593 | -0.036 | 0.418 | 0.028 | 0.786 | -0.113 |
| 50-300 | 0.255 | 0.006 | 0.465 | -0.028 | 0.315 | 0.025 | 0.639 | -0.070 |
| 50-400 | 0.212 | -0.001 | 0.368 | -0.023 | 0.296 | 0.023 | 0.564 | -0.045 |
| 50-500 | 0.190 | 0.001 | 0.326 | -0.016 | 0.249 | 0.016 | 0.472 | -0.037 |
| 50-1000 | 0.131 | -0.012 | 0.220 | -0.006 | 0.163 | 0.014 | 0.297 | -0.012 |
| 50-5000 | 0.058 | -0.012 | 0.095 | -0.002 | 0.065 | 0.001 | 0.115 | -0.001 |

**Table S3.** Item Recovery Results for C-RUM Model with 36 Items

|  | 3 Attributes | | | | 5 Attributes | | | |
| --- | --- | --- | --- | --- | --- | --- | --- | --- |
|  | Intercept | | Main | | Intercept | | Main | |
|  | RMSE | BIAS | RMSE | BIAS | RMSE | BIAS | RMSE | BIAS |
| 25-50 | 0.477 | 0.046 | 0.981 | -0.064 | 0.578 | 0.037 | 1.100 | -0.236 |
| 25-100 | 0.329 | 0.018 | 0.811 | -0.063 | 0.421 | 0.020 | 0.918 | -0.154 |
| 25-200 | 0.215 | -0.002 | 0.596 | -0.045 | 0.297 | 0.020 | 0.711 | -0.070 |
| 25-300 | 0.173 | -0.001 | 0.445 | -0.036 | 0.228 | 0.018 | 0.598 | -0.035 |
| 25-400 | 0.146 | -0.001 | 0.372 | -0.026 | 0.193 | 0.013 | 0.512 | -0.018 |
| 25-500 | 0.131 | -0.001 | 0.327 | -0.020 | 0.169 | 0.006 | 0.459 | -0.013 |
| 25-1000 | 0.093 | -0.001 | 0.228 | -0.016 | 0.115 | 0.004 | 0.173 | -0.008 |
| 25-5000 | 0.040 | -0.001 | 0.096 | -0.008 | 0.051 | 0.001 | 0.118 | -0.002 |
| 50-50 | 0.618 | 0.044 | 0.969 | -0.160 | 0.594 | 0.041 | 1.060 | -0.297 |
| 50-100 | 0.472 | 0.045 | 0.735 | -0.086 | 0.512 | 0.034 | 0.887 | -0.173 |
| 50-200 | 0.290 | 0.008 | 0.469 | -0.028 | 0.343 | 0.025 | 0.630 | -0.071 |
| 50-300 | 0.225 | 0.002 | 0.364 | -0.016 | 0.264 | 0.025 | 0.493 | -0.045 |
| 50-400 | 0.190 | -0.002 | 0.302 | -0.010 | 0.222 | 0.025 | 0.403 | -0.028 |
| 50-500 | 0.174 | -0.002 | 0.273 | -0.008 | 0.192 | 0.014 | 0.341 | -0.022 |
| 50-1000 | 0.117 | -0.002 | 0.185 | -0.007 | 0.129 | 0.011 | 0.224 | -0.012 |
| 50-5000 | 0.053 | -0.007 | 0.080 | -0.002 | 0.057 | 0.002 | 0.095 | -0.002 |

**Table S4.** Item Recovery Results for DINA Model with 12 Items

|  | 3 Attributes | | | | 5 Attributes | | | |
| --- | --- | --- | --- | --- | --- | --- | --- | --- |
|  | Intercept | | e parameter | | Intercept | | e parameter | |
|  | RMSE | BIAS | RMSE | BIAS | RMSE | BIAS | RMSE | BIAS |
| 25-50 | 0.532 | 0.093 | 1.067 | 0.592 | 0.608 | 0.183 | 0.887 | -0.085 |
| 25-100 | 0.365 | 0.032 | 0.827 | 0.214 | 0.542 | 0.218 | 0.772 | -0.231 |
| 25-200 | 0.250 | -0.005 | 0.664 | 0.025 | 0.400 | 0.147 | 0.587 | -0.191 |
| 25-300 | 0.203 | -0.016 | 0.589 | -0.023 | 0.362 | 0.124 | 0.494 | -0.158 |
| 25-400 | 0.180 | -0.020 | 0.524 | -0.025 | 0.345 | 0.119 | 0.439 | -0.146 |
| 25-500 | 0.161 | -0.021 | 0.469 | 0.004 | 0.351 | 0.113 | 0.416 | -0.121 |
| 25-1000 | 0.119 | -0.023 | 0.343 | 0.032 | 0.287 | 0.088 | 0.329 | -0.089 |
| 25-5000 | 0.068 | -0.025 | 0.170 | 0.067 | 0.141 | 0.023 | 0.175 | -0.026 |
| 50-50 | 0.626 | 0.031 | 0.841 | 0.133 | 0.628 | 0.134 | 0.926 | 0.057 |
| 50-100 | 0.462 | 0.005 | 0.710 | -0.014 | 0.531 | 0.151 | 0.800 | -0.153 |
| 50-200 | 0.319 | -0.016 | 0.530 | -0.001 | 0.435 | 0.122 | 0.632 | -0.154 |
| 50-300 | 0.253 | -0.036 | 0.439 | 0.011 | 0.397 | 0.110 | 0.575 | -0.156 |
| 50-400 | 0.232 | -0.040 | 0.373 | 0.025 | 0.366 | 0.097 | 0.510 | -0.132 |
| 50-500 | 0.204 | -0.040 | 0.333 | 0.043 | 0.369 | 0.099 | 0.464 | -0.123 |
| 50-1000 | 0.155 | -0.042 | 0.247 | 0.048 | 0.246 | 0.048 | 0.347 | -0.065 |
| 50-5000 | 0.100 | -0.044 | 0.134 | 0.060 | 0.101 | 0.007 | 0.149 | -0.015 |

**Table S5.** Item Recovery Results for DINA Model with 24 Items

|  | 3 Attributes | | | | 5 Attributes | | | |
| --- | --- | --- | --- | --- | --- | --- | --- | --- |
|  | Intercept | | e parameter | | Intercept | | e parameter | |
|  | RMSE | BIAS | RMSE | BIAS | RMSE | BIAS | RMSE | BIAS |
| 25-50 | 0.424 | 0.051 | 0.924 | 0.461 | 0.537 | 0.121 | 0.792 | -0.08 |
| 25-100 | 0.271 | 0.001 | 0.702 | 0.086 | 0.375 | 0.060 | 0.631 | -0.116 |
| 25-200 | 0.189 | -0.001 | 0.613 | -0.009 | 0.249 | 0.031 | 0.458 | -0.071 |
| 25-300 | 0.152 | 0.001 | 0.528 | -0.014 | 0.196 | 0.025 | 0.368 | -0.054 |
| 25-400 | 0.134 | -0.001 | 0.472 | 0.003 | 0.170 | 0.018 | 0.303 | -0.037 |
| 25-500 | 0.123 | 0.001 | 0.429 | 0.015 | 0.158 | 0.017 | 0.274 | -0.032 |
| 25-1000 | 0.086 | -0.012 | 0.322 | 0.034 | 0.110 | 0.001 | 0.189 | -0.009 |
| 25-5000 | 0.040 | -0.008 | 0.179 | 0.060 | 0.047 | 0.001 | 0.085 | 0.001 |
| 50-50 | 0.513 | 0.043 | 0.791 | 0.017 | 0.545 | 0.108 | 0.813 | -0.025 |
| 50-100 | 0.349 | 0.002 | 0.630 | -0.011 | 0.398 | 0.052 | 0.659 | -0.103 |
| 50-200 | 0.232 | -0.004 | 0.462 | 0.017 | 0.253 | 0.030 | 0.474 | -0.076 |
| 50-300 | 0.184 | -0.006 | 0.382 | 0.024 | 0.201 | 0.025 | 0.388 | -0.056 |
| 50-400 | 0.164 | -0.009 | 0.325 | 0.041 | 0.176 | 0.017 | 0.324 | -0.043 |
| 50-500 | 0.152 | -0.010 | 0.301 | 0.048 | 0.159 | 0.009 | 0.289 | -0.029 |
| 50-1000 | 0.106 | -0.020 | 0.227 | 0.061 | 0.111 | 0.001 | 0.200 | -0.009 |
| 50-5000 | 0.053 | -0.018 | 0.134 | 0.068 | 0.048 | 0.001 | 0.089 | -0.001 |

**Table S6.** Item Recovery Results for DINA Model with 36 Items

|  | 3 Attributes | | | | 5 Attributes | | | |
| --- | --- | --- | --- | --- | --- | --- | --- | --- |
|  | Intercept | | e parameter | | Intercept | | e parameter | |
|  | RMSE | BIAS | RMSE | BIAS | RMSE | BIAS | RMSE | BIAS |
| 25-50 | 0.406 | 0.035 | 0.851 | 0.351 | 0.518 | 0.083 | 0.778 | -0.092 |
| 25-100 | 0.275 | 0.010 | 0.706 | 0.029 | 0.351 | 0.042 | 0.612 | -0.118 |
| 25-200 | 0.191 | 0.003 | 0.602 | -0.035 | 0.233 | 0.018 | 0.440 | -0.070 |
| 25-300 | 0.154 | 0.001 | 0.522 | -0.023 | 0.188 | 0.009 | 0.347 | -0.043 |
| 25-400 | 0.132 | -0.001 | 0.439 | -0.003 | 0.161 | 0.006 | 0.294 | -0.032 |
| 25-500 | 0.118 | -0.001 | 0.392 | 0.004 | 0.145 | 0.007 | 0.263 | -0.026 |
| 25-1000 | 0.083 | -0.004 | 0.290 | 0.029 | 0.101 | 0.003 | 0.184 | -0.012 |
| 25-5000 | 0.037 | -0.003 | 0.156 | 0.048 | 0.044 | 0.002 | 0.079 | -0.003 |
| 50-50 | 0.497 | 0.047 | 0.772 | -0.036 | 0.522 | 0.074 | 0.790 | -0.064 |
| 50-100 | 0.333 | 0.019 | 0.611 | -0.057 | 0.352 | 0.035 | 0.628 | -0.114 |
| 50-200 | 0.227 | 0.008 | 0.445 | -0.016 | 0.235 | 0.015 | 0.453 | -0.068 |
| 50-300 | 0.186 | 0.003 | 0.363 | 0.007 | 0.188 | 0.010 | 0.356 | -0.043 |
| 50-400 | 0.157 | 0.001 | 0.311 | 0.018 | 0.162 | 0.006 | 0.303 | -0.034 |
| 50-500 | 0.141 | 0.002 | 0.283 | 0.024 | 0.145 | 0.006 | 0.266 | -0.026 |
| 50-1000 | 0.099 | -0.001 | 0.214 | 0.035 | 0.101 | 0.003 | 0.186 | -0.014 |
| 50-5000 | 0.044 | -0.004 | 0.118 | 0.045 | 0.045 | 0.002 | 0.080 | -0.003 |

**Table S7.** Item Recovery Results for DINO Model with 12 Items

|  | 3 Attributes | | | | 5 Attributes | | | |
| --- | --- | --- | --- | --- | --- | --- | --- | --- |
|  | Intercept | | e parameter | | Intercept | | e parameter | |
|  | RMSE | BIAS | RMSE | BIAS | RMSE | BIAS | RMSE | BIAS |
| 25-50 | 0.491 | 0.069 | 0.905 | 0.269 | 0.632 | 0.136 | 0.891 | 0.1777 |
| 25-100 | 0.304 | -0.011 | 0.710 | 0.115 | 0.433 | 0.062 | 0.689 | -0.172 |
| 25-200 | 0.208 | -0.010 | 0.600 | -0.114 | 0.291 | 0.011 | 0.602 | -0.168 |
| 25-300 | 0.168 | -0.009 | 0.516 | -0.114 | 0.241 | 0.018 | 0.502 | -0.132 |
| 25-400 | 0.150 | -0.008 | 0.466 | -0.111 | 0.202 | -0.001 | 0.449 | -0.126 |
| 25-500 | 0.129 | -0.005 | 0.406 | -0.085 | 0.177 | -0.004 | 0.407 | -0.106 |
| 25-1000 | 0.093 | -0.003 | 0.283 | -0.052 | 0.126 | -0.012 | 0.326 | -0.081 |
| 25-5000 | 0.041 | -0.003 | 0.122 | -0.027 | 0.056 | -0.003 | 0.210 | -0.052 |
| 50-50 | 0.619 | 0.026 | 0.878 | 0.083 | 0.688 | 0.182 | 0.934 | 0.118 |
| 50-100 | 0.438 | 0.021 | 0.677 | -0.083 | 0.567 | 0.108 | 0.754 | -0.091 |
| 50-200 | 0.304 | 0.021 | 0.500 | -0.059 | 0.357 | 0.021 | 0.618 | -0.088 |
| 50-300 | 0.249 | -0.027 | 0.396 | -0.026 | 0.288 | 0.011 | 0.523 | -0.057 |
| 50-400 | 0.212 | -0.018 | 0.342 | -0.024 | 0.241 | -0.001 | 0.462 | -0.042 |
| 50-500 | 0.203 | -0.022 | 0.307 | -0.014 | 0.216 | 0.002 | 0.444 | -0.061 |
| 50-1000 | 0.143 | -0.021 | 0.222 | -0.016 | 0.151 | -0.002 | 0.349 | -0.034 |
| 50-5000 | 0.064 | -0.020 | 0.095 | -0.006 | 0.068 | -0.001 | 0.187 | -0.021 |

**Table S8.** Item Recovery Results for DINO Model with 24 Items

|  | 3 Attributes | | | | 5 Attributes | | | |
| --- | --- | --- | --- | --- | --- | --- | --- | --- |
|  | Intercept | | e parameter | | Intercept | | e parameter | |
|  | RMSE | BIAS | RMSE | BIAS | RMSE | BIAS | RMSE | BIAS |
| 25-50 | 0.425 | 0.051 | 0.757 | 0.080 | 0.552 | 0.073 | 0.784 | -0.086 |
| 25-100 | 0.287 | 0.013 | 0.663 | -0.087 | 0.381 | 0.041 | 0.623 | -0.082 |
| 25-200 | 0.196 | 0.009 | 0.500 | -0.075 | 0.255 | 0.025 | 0.447 | -0.079 |
| 25-300 | 0.159 | 0.008 | 0.415 | -0.063 | 0.204 | 0.018 | 0.365 | -0.058 |
| 25-400 | 0.141 | 0.006 | 0.348 | -0.005 | 0.177 | 0.012 | 0.303 | -0.041 |
| 25-500 | 0.126 | 0.007 | 0.306 | -0.004 | 0.163 | 0.012 | 0.273 | -0.034 |
| 25-1000 | 0.092 | 0.002 | 0.231 | -0.010 | 0.112 | 0.007 | 0.186 | -0.011 |
| 25-5000 | 0.052 | -0.001 | 0.077 | -0.007 | 0.049 | 0.001 | 0.085 | -0.002 |
| 50-50 | 0.557 | 0.049 | 0.779 | -0.094 | 0.607 | 0.073 | 0.833 | -0.142 |
| 50-100 | 0.405 | 0.035 | 0.596 | -0.097 | 0.438 | 0.049 | 0.657 | -0.117 |
| 50-200 | 0.263 | 0.015 | 0.419 | -0.054 | 0.286 | 0.028 | 0.469 | -0.074 |
| 50-300 | 0.208 | 0.013 | 0.333 | -0.044 | 0.228 | 0.018 | 0.378 | -0.053 |
| 50-400 | 0.182 | 0.006 | 0.286 | -0.032 | 0.194 | 0.010 | 0.317 | -0.036 |
| 50-500 | 0.168 | 0.005 | 0.259 | -0.025 | 0.180 | 0.009 | 0.285 | -0.024 |
| 50-1000 | 0.116 | -0.002 | 0.178 | -0.012 | 0.123 | 0.003 | 0.196 | -0.014 |
| 50-5000 | 0.052 | -0.001 | 0.077 | -0.007 | 0.054 | 0.001 | 0.086 | -0.002 |

**Table S9.** Item Recovery Results for DINO Model with 36 Items

|  | 3 Attributes | | | | 5 Attributes | | | |
| --- | --- | --- | --- | --- | --- | --- | --- | --- |
|  | Intercept | | e parameter | | Intercept | | e parameter | |
|  | RMSE | BIAS | RMSE | BIAS | RMSE | BIAS | RMSE | BIAS |
| 25-50 | 0.424 | 0.034 | 0.749 | 0.152 | 0.543 | 0.063 | 0.792 | -0.121 |
| 25-100 | 0.289 | 0.014 | 0.647 | -0.102 | 0.365 | 0.031 | 0.607 | -0.117 |
| 25-200 | 0.199 | 0.008 | 0.482 | -0.081 | 0.247 | 0.018 | 0.426 | -0.068 |
| 25-300 | 0.162 | 0.005 | 0.397 | -0.062 | 0.199 | 0.010 | 0.334 | -0.041 |
| 25-400 | 0.139 | 0.004 | 0.329 | -0.044 | 0.170 | 0.008 | 0.284 | -0.030 |
| 25-500 | 0.124 | 0.005 | 0.293 | -0.036 | 0.153 | 0.008 | 0.254 | -0.023 |
| 25-1000 | 0.087 | 0.003 | 0.203 | -0.022 | 0.107 | 0.004 | 0.176 | -0.009 |
| 25-5000 | 0.038 | 0.001 | 0.088 | -0.007 | 0.047 | 0.002 | 0.076 | -0.002 |
| 50-50 | 0.583 | 0.068 | 0.775 | -0.111 | 0.595 | 0.073 | 0.818 | -0.111 |
| 50-100 | 0.385 | 0.032 | 0.581 | -0.103 | 0.408 | 0.043 | 0.622 | -0.113 |
| 50-200 | 0.259 | 0.016 | 0.406 | -0.063 | 0.269 | 0.023 | 0.427 | -0.064 |
| 50-300 | 0.211 | 0.007 | 0.322 | -0.037 | 0.216 | 0.014 | 0.347 | -0.041 |
| 50-400 | 0.178 | 0.006 | 0.274 | -0.030 | 0.184 | 0.012 | 0.291 | -0.031 |
| 50-500 | 0.162 | 0.006 | 0.248 | -0.025 | 0.164 | 0.011 | 0.256 | -0.026 |
| 50-1000 | 0.113 | 0.003 | 0.171 | -0.013 | 0.115 | 0.007 | 0.179 | -0.013 |
| 50-5000 | 0.049 | -0.001 | 0.076 | -0.004 | 0.051 | 0.002 | 0.077 | -0.003 |

**Table S10.** Item Recovery Results for LCDMREDUCED Model with 12 Items

|  | 3 ATT | | | | | | | | 5 ATT | | | | | | | |
| --- | --- | --- | --- | --- | --- | --- | --- | --- | --- | --- | --- | --- | --- | --- | --- | --- |
|  | Intercept | | Main | | e parameter | | Interaction | | Intercept | | Main | | e parameter | | Interaction | |
|  | RMSE | BIAS | RMSE | BIAS | RMSE | BIAS | RMSE | BIAS | RMSE | BIAS | RMSE | BIAS | RMSE | BIAS | RMSE | BIAS |
| 25-50 | 0.596 | 0.181 | 1.072 | 0.084 | 1.266 | 0.718 | 1.389 | -0.601 | 0.734 | 0.193 | 1.206 | -0.059 | 0.936 | -0.060 | 1.239 | 0.214 |
| 25-100 | 0.434 | 0.104 | 0.968 | -0.009 | 0.873 | 0.270 | 1.317 | -0.567 | 0.613 | 0.194 | 1.147 | -0.215 | 0.770 | -0.187 | 1.211 | 0.414 |
| 25-200 | 0.265 | 0.016 | 0.762 | -0.006 | 0.756 | 0.0137 | 1.313 | -0.668 | 0.369 | 0.068 | 0.995 | -0.180 | 0.615 | -0.168 | 1.488 | 0.445 |
| 25-300 | 0.199 | 0.003 | 0.617 | -0.035 | 0.670 | -0.049 | 1.251 | -0.706 | 0.360 | 0.093 | 0.921 | -0.179 | 0.513 | -0.161 | 1.334 | 0.049 |
| 25-400 | 0.168 | -0.006 | 0.529 | -0.036 | 0.606 | -0.072 | 1.225 | -0.719 | 0.287 | 0.052 | 0.868 | -0.158 | 0.427 | -0.086 | 1.436 | -0.014 |
| 25-500 | 0.149 | -0.008 | 0.439 | -0.019 | 0.546 | -0.061 | 1.182 | -0.731 | 0.268 | 0.054 | 0.799 | -0.128 | 0.408 | -0.008 | 1.452 | -0.021 |
| 25-1000 | 0.102 | -0.011 | 0.294 | -0.011 | 0.423 | -0.070 | 1.044 | -0.750 | 0.180 | 0.026 | 0.658 | -0.094 | 0.275 | -0.050 | 1.368 | -0.089 |
| 25-5000 | 0.047 | -0.007 | 0.126 | -0.010 | 0.168 | -0.037 | 0.830 | -0.701 | 0.072 | 0.002 | 0.277 | -0.022 | 0.115 | -0.007 | 0.923 | 0.042 |
| 50-50 | 0.672 | 0.083 | 1.021 | -0.056 | 0.951 | 0.039 | 1.344 | -0.555 | 0.741 | 0.104 | 1.181 | -0.028 | 1.008 | 0.194 | 1.242 | 0.256 |
| 50-100 | 0.527 | 0.046 | 0.858 | -0.099 | 0.811 | -0.047 | 1.444 | -0.694 | 0.589 | 0.111 | 1.144 | -0.201 | 0.789 | 0.002 | 1.454 | 0.621 |
| 50-200 | 0.350 | 0.023 | 0.612 | -0.062 | 0.620 | -0.104 | 1.269 | -0.709 | 0.508 | 0.084 | 0.972 | -0.191 | 0.665 | -0.122 | 1.393 | 0.115 |
| 50-300 | 0.276 | 0.0001 | 0.492 | -0.062 | 0.515 | -0.088 | 1.173 | -0.643 | 0.432 | 0.083 | 0.905 | -0.211 | 0.588 | -0.136 | 1.554 | 0.422 |
| 50-400 | 0.238 | -0.003 | 0.407 | -0.031 | 0.440 | -0.047 | 1.095 | -0.687 | 0.339 | 0.0454 | 0.808 | -0.176 | 0.525 | -0.097 | 1.473 | 0.350 |
| 50-500 | 0.209 | -0.009 | 0.354 | -0.011 | 0.395 | -0.043 | 1.024 | -0.710 | 0.331 | 0.053 | 0.753 | -0.143 | 0.459 | -0.080 | 1.403 | 0.059 |
| 50-1000 | 0.149 | -0.007 | 0.255 | -0.015 | 0.294 | -0.036 | 0.935 | -0.672 | 0.214 | 0.020 | 0.550 | -0.099 | 0.340 | -0.054 | 1.313 | 0.162 |
| 50-5000 | 0.065 | -0.016 | 0.108 | -0.004 | 0.122 | -0.005 | 0.795 | -0.684 | 0.085 | 0.001 | 0.200 | -0.015 | 0.136 | -0.013 | 0.587 | 0.002 |

**Table S11.** Item Recovery Results for LCDMREDUCED Model with 24 Items

|  | 3 ATT | | | | | | | | 5 ATT | | | | | | | |
| --- | --- | --- | --- | --- | --- | --- | --- | --- | --- | --- | --- | --- | --- | --- | --- | --- |
|  | Intercept | | Main | | e parameter | | Interaction | | Intercept | | Main | | e parameter | | Interaction | |
|  | RMSE | BIAS | RMSE | BIAS | RMSE | BIAS | RMSE | BIAS | RMSE | BIAS | RMSE | BIAS | RMSE | BIAS | RMSE | BIAS |
| 25-50 | 0.449 | 0.080 | 0.996 | -0.065 | 0.920 | 0.333 | 1.428 | 0.396 | 0.602 | 0.127 | 1.135 | -0.192 | 0.851 | -0.055 | 1.519 | 0.360 |
| 25-100 | 0.298 | 0.016 | 0.766 | -0.106 | 0.707 | 0.020 | 1.401 | 0.332 | 0.421 | 0.065 | 0.993 | -0.213 | 0.672 | -0.165 | 1.569 | 0.437 |
| 25-200 | 0.200 | 0.006 | 0.557 | -0.074 | 0.590 | -0.082 | 1.119 | 0.079 | 0.298 | 0.052 | 0.801 | -0.144 | 0.473 | -0.072 | 1.465 | 0.264 |
| 25-300 | 0.160 | 0.006 | 0.429 | -0.063 | 0.535 | -0.088 | 0.931 | -0.002 | 0.224 | 0.032 | 0.670 | -0.106 | 0.374 | -0.058 | 1.490 | 0.281 |
| 25-400 | 0.140 | 0.005 | 0.368 | -0.050 | 0.449 | -0.073 | 0.852 | -0.024 | 0.196 | 0.020 | 0.562 | -0.064 | 0.324 | -0.045 | 1.303 | 0.568 |
| 25-500 | 0.127 | 0.006 | 0.326 | -0.049 | 0.400 | -0.066 | 0.739 | 0.016 | 0.177 | 0.020 | 0.521 | -0.035 | 0.285 | -0.030 | 1.285 | -0.083 |
| 25-1000 | 0.091 | -0.004 | 0.238 | -0.041 | 0.276 | -0.037 | 0.495 | 0.013 | 0.118 | 0.004 | 0.351 | -0.021 | 0.194 | -0.005 | 0.898 | 0.031 |
| 25-5000 | 0.041 | -0.004 | 0.114 | -0.036 | 0.119 | -0.014 | 0.221 | 0.063 | 0.052 | 0.003 | 0.135 | -0.002 | 0.085 | -0.002 | 0.360 | 0.006 |
| 50-50 | 0.582 | 0.083 | 0.936 | -0.125 | 0.810 | -0.033 | 1.280 | -0.016 | 0.623 | 0.074 | 1.090 | -0.109 | 0.846 | -0.007 | 1.552 | 0.190 |
| 50-100 | 0.406 | 0.032 | 0.716 | -0.133 | 0.666 | -0.108 | 1.315 | 0.344 | 0.483 | 0.066 | 0.929 | -0.179 | 0.684 | -0.118 | 1.499 | 0.352 |
| 50-200 | 0.265 | 0.017 | 0.458 | -0.064 | 0.469 | -0.059 | 0.864 | 0.032 | 0.340 | 0.042 | 0.726 | -0.124 | 0.515 | -0.097 | 1.389 | 0.185 |
| 50-300 | 0.211 | 0.015 | 0.358 | -0.059 | 0.371 | -0.044 | 0.719 | 0.037 | 0.252 | 0.021 | 0.569 | -0.072 | 0.430 | -0.057 | 1.281 | 0.102 |
| 50-400 | 0.185 | 0.014 | 0.309 | -0.058 | 0.325 | -0.048 | 0.675 | 0.007 | 0.219 | 0.020 | 0.491 | -0.046 | 0.365 | -0.045 | 1.126 | -0.007 |
| 50-500 | 0.165 | 0.008 | 0.285 | -0.047 | 0.294 | -0.034 | 0.574 | -0.034 | 0.202 | 0.008 | 0.437 | -0.037 | 0.333 | -0.030 | 1.104 | 0.095 |
| 50-1000 | 0.116 | -0.003 | 0.205 | -0.046 | 0.195 | -0.005 | 0.392 | 0.030 | 0.136 | 0.018 | 0.287 | -0.014 | 0.226 | -0.011 | 0.725 | 0.031 |
| 50-5000 | 0.054 | -0.003 | 0.111 | -0.039 | 0.087 | -0.004 | 0.172 | 0.046 | 0.063 | 0.001 | 0.135 | 0.003 | 0.130 | 0.011 | 0.324 | -0.010 |

**Table S12.** Item Recovery Results for LCDMREDUCED Model with 36 Items

|  | 3 ATT | | | | | | | | 5 ATT | | | | | | | |
| --- | --- | --- | --- | --- | --- | --- | --- | --- | --- | --- | --- | --- | --- | --- | --- | --- |
|  | Intercept | | Main | | e parameter | | Interaction | | Intercept | | Main | | e parameter | | Interaction | |
|  | RMSE | BIAS | RMSE | BIAS | RMSE | BIAS | RMSE | BIAS | RMSE | BIAS | RMSE | BIAS | RMSE | BIAS | RMSE | BIAS |
| 25-50 | 0.451 | 0.075 | 0.922 | -0.067 | 0.888 | 0.288 | 1.271 | 0.320 | 0.563 | 0.096 | 1.077 | -0.232 | 0.786 | -0.089 | 1.528 | 0.261 |
| 25-100 | 0.313 | 0.035 | 0.707 | -0.053 | 0.699 | 0.048 | 1.236 | -0.007 | 0.396 | 0.052 | 0.889 | -0.142 | 0.623 | -0.086 | 1.518 | 0.187 |
| 25-200 | 0.251 | 0.043 | 0.522 | -0.019 | 0.629 | 0.019 | 1.041 | -0.028 | 0.264 | 0.018 | 0.661 | -0.065 | 0.431 | -0.056 | 1.357 | 0.118 |
| 25-300 | 0.219 | 0.066 | 0.433 | -0.001 | 0.548 | 0.078 | 1.010 | -0.290 | 0.211 | 0.014 | 0.535 | -0.048 | 0.338 | -0.044 | 1.272 | 0.086 |
| 25-400 | 0.196 | 0.059 | 0.395 | 0.008 | 0.501 | 0.089 | 0.872 | -0.314 | 0.180 | 0.015 | 0.462 | -0.021 | 0.296 | -0.032 | 1.033 | -0.104 |
| 25-500 | 0.178 | 0.061 | 0.359 | 0.012 | 0.463 | 0.097 | 0.786 | -0.291 | 0.159 | 0.009 | 0.417 | -0.015 | 0.262 | -0.022 | 1.000 | -0.024 |
| 25-1000 | 0.168 | 0.074 | 0.309 | 0.021 | 0.409 | 0.159 | 0.640 | -0.340 | 0.109 | 0.004 | 0.268 | -0.008 | 0.183 | -0.010 | 0.642 | 0.002 |
| 25-5000 | 0.105 | 0.056 | 0.190 | 0.021 | 0.265 | 0.146 | 0.434 | -0.261 | 0.047 | 0.001 | 0.113 | -0.002 | 0.078 | -0.004 | 0.253 | 0.012 |
| 50-50 | 0.569 | 0.094 | 0.892 | -0.181 | 0.787 | -0.094 | 1.381 | 0.270 | 0.590 | 0.060 | 1.019 | -0.179 | 0.811 | -0.022 | 1.371 | 0.371 |
| 50-100 | 0.374 | 0.030 | 0.629 | -0.114 | 0.604 | -0.090 | 1.170 | 0.141 | 0.433 | 0.044 | 0.849 | -0.159 | 0.640 | -0.105 | 1.476 | 0.405 |
| 50-200 | 0.251 | 0.018 | 0.420 | -0.082 | 0.442 | -0.065 | 0.808 | 0.093 | 0.291 | 0.029 | 0.581 | -0.078 | 0.457 | -0.075 | 1.254 | 0.209 |
| 50-300 | 0.206 | 0.012 | 0.343 | -0.065 | 0.347 | -0.039 | 0.654 | 0.077 | 0.237 | 0.025 | 0.468 | -0.042 | 0.362 | -0.050 | 1.058 | 0.076 |
| 50-400 | 0.175 | 0.011 | 0.290 | -0.056 | 0.296 | -0.031 | 0.547 | 0.048 | 0.198 | 0.023 | 0.383 | -0.027 | 0.309 | -0.035 | 0.867 | 0.011 |
| 50-500 | 0.156 | 0.011 | 0.266 | -0.054 | 0.264 | -0.025 | 0.492 | 0.037 | 0.168 | 0.011 | 0.339 | -0.015 | 0.272 | -0.028 | 0.794 | -0.035 |
| 50-1000 | 0.109 | 0.006 | 0.196 | -0.049 | 0.182 | -0.012 | 0.342 | 0.044 | 0.116 | 0.006 | 0.229 | -0.008 | 0.187 | -0.014 | 0.557 | -0.019 |
| 50-5000 | 0.052 | 0.002 | 0.106 | -0.041 | 0.079 | -0.001 | 0.158 | 0.036 | 0.052 | 0.001 | 0.096 | -0.002 | 0.080 | **-0.003** | 0.220 | 0.005 |

**Table S13.** Classification Accuracy Percentages

|  | N | 3ATT12ITEM25BR | 5ATT12ITEM25BR | 3ATT24ITEM25BR | 5ATT24ITEM25BR | 3ATT36ITEM25BR | 5ATT36ITEM25BR | 3ATT12ITEM50BR | 5ATT12ITEM50BR | 3ATT24ITEM50BR | 5ATT24ITEM50BR | 3ATT36ITEM50BR | 5ATT36ITEM50BR |
| --- | --- | --- | --- | --- | --- | --- | --- | --- | --- | --- | --- | --- | --- |
|  | 50 | 45.02 | 29.02 | 57.66 | 56.02 | 59.58 | 57.88 | 45.84 | 21.64 | 53.22 | 55.46 | 55.12 | 55.37 |
|  | 100 | 50.9 | 29.8 | 58.01 | 56.32 | 58.68 | 57.01 | 47.36 | 22.86 | 53.89 | 55.55 | 54.51 | 55.76 |
|  | 200 | 58.455 | 34.97 | 58.26 | 56.965 | 58.24 | 56.885 | 51.435 | 26.135 | 54.995 | 55.8 | 54.2 | 55.865 |
| C-RUM | 300 | 61.39 | 38.946 | 58.81 | 56.846 | 58.037 | 57.06 | 53.93 | 27.116 | 54.193 | 56.08 | 54.296 | 55.947 |
|  | 400 | 64.745 | 40.525 | 57.605 | 56.725 | 57.82 | 56.73 | 55.047 | 28.112 | 53.695 | 55.605 | 53.958 | 55.785 |
|  | 500 | 64.798 | 42.372 | 57.62 | 56.786 | 57.734 | 56.582 | 55.572 | 30.248 | 53.612 | 55.452 | 53.894 | 55.584 |
|  | 1000 | 68.623 | 47.767 | 57.938 | 56.854 | 57.851 | 56.666 | 59.026 | 34.114 | 54.018 | 55.672 | 53.901 | 55.583 |
|  | 5000 | 70.344 | 62.935 | 57.94 | 56.741 | 57.846 | 56.722 | 62.226 | 44.912 | 54.112 | 55.61 | 54.057 | 55.587 |
|  | 50 | 69.04 | 53.38 | 57.82 | 65.34 | 59 | 66.52 | 73.4 | 47.48 | 56.32 | 63.74 | 58.58 | 64.8 |
|  | 100 | 73.72 | 55.16 | 57.78 | 66 | 58.8 | 66.38 | 74.83 | 49.29 | 57.73 | 64.49 | 58.37 | 64.95 |
|  | 200 | 76.2 | 59.25 | 57.595 | 66.305 | 58.19 | 66.095 | 77.755 | 51.705 | 58.2 | 64.45 | 57.845 | 64.42 |
| DINA | 300 | 77.073 | 59.787 | 57.603 | 65.96 | 57.837 | 66.227 | 78.333 | 52.67 | 57.67 | 64.16 | 57.77 | 64.38 |
|  | 400 | 77.253 | 61.44 | 57.463 | 65.838 | 57.673 | 66.03 | 78.388 | 53.715 | 56.957 | 63.873 | 57.648 | 64.155 |
|  | 500 | 77.474 | 61.736 | 57.356 | 65.866 | 57.722 | 66.028 | 78.726 | 53.308 | 57.156 | 63.962 | 57.622 | 64.112 |
|  | 1000 | 77.732 | 63.257 | 57.468 | 66.046 | 57.436 | 66.052 | 79.753 | 54.632 | 57.407 | 64.236 | 57.612 | 64.163 |
|  | 5000 | 77.777 | 69.168 | 57.552 | 66.073 | 57.455 | 66.115 | 80.21 | 57.975 | 57.513 | 64.177 | 57.6 | 64.174 |
|  | 50 | 81.8 | 62.64 | 59.32 | 65.92 | 60.92 | 68.24 | 76.66 | 51 | 58.58 | 64.52 | 61.04 | 67.56 |
|  | 100 | 84.99 | 64.57 | 58.81 | 67.5 | 59.8 | 67.61 | 77.97 | 54.05 | 60.35 | 66.66 | 60.84 | 66.8 |
|  | 200 | 86.23 | 66.58 | 58.835 | 67.32 | 59.44 | 67.16 | 79.775 | 55.92 | 60.67 | 66.26 | 60.23 | 66.44 |
| DINO | 300 | 86.813 | 67.897 | 58.83 | 67.05 | 59.07 | 67.38 | 80.17 | 55.977 | 60.207 | 66.183 | 60.167 | 66.38 |
|  | 400 | 87.003 | 69.302 | 58.47 | 66.907 | 58.94 | 67.378 | 80.9 | 57 | 59.515 | 65.835 | 60.13 | 66.345 |
|  | 500 | 87.278 | 69.752 | 58.7 | 66.866 | 58.814 | 67.33 | 81.01 | 57.44 | 59.654 | 65.826 | 60.108 | 66.226 |
|  | 1000 | 87.963 | 70.838 | 58.735 | 67.061 | 58.803 | 67.338 | 81.649 | 58.622 | 59.909 | 66.02 | 60.099 | 66.236 |
|  | 5000 | 88.272 | 74.422 | 59.988 | 67.107 | 58.67 | 67.255 | 81.912 | 59.86 | 59.988 | 66.972 | 60.089 | 66.148 |
|  | 50 | 61.96 | 40.46 | 57.8 | 56.14 | 58.62 | 56.66 | 65.96 | 32.82 | 53.76 | 55.18 | 55.72 | 55.48 |
|  | 100 | 68.45 | 47.15 | 58.23 | 56.78 | 58.62 | 56.73 | 68.18 | 36.69 | 54.08 | 56.42 | 54.43 | 55.76 |
|  | 200 | 73.05 | 51.575 | 58.25 | 56.605 | 58.405 | 57.435 | 69.96 | 39.13 | 54.445 | 55.465 | 54.355 | 55.995 |
| LCDMREDUCED | 300 | 75.15 | 55.403 | 57.963 | 56.703 | 58.423 | 56.81 | 71.24 | 40.48 | 53.86 | 55.397 | 54.403 | 56.03 |
|  | 400 | 75.875 | 55.415 | 57.713 | 57.29 | 58.305 | 56.95 | 71.815 | 43.615 | 54.248 | 55.663 | 54.01 | 55.662 |
|  | 500 | 76.392 | 58.352 | 57.692 | 57.068 | 58.084 | 56.854 | 72.198 | 44.316 | 54.266 | 55.788 | 53.81 | 55.736 |
|  | 1000 | 77.289 | 61.073 | 57.969 | 56.991 | 57.565 | 56.764 | 73.105 | 46.904 | 54.018 | 55.709 | 53.861 | 55.714 |
|  | 5000 | 77.456 | 68.207 | 57.87 | 56.881 | 57.857 | 56.836 | 74.049 | 52.031 | 54.102 | 55.75 | 54.11 | 55.693 |
